# Dynamic Coagulation Deterioration and Cumulative Burden Preceding Noncoagulation Organ Dysfunction in Sepsis: A Two-Database Cohort Study

**DOI:** 10.3390/jcm15218326

**Published:** 2026-10-26

**Authors:** Xiang Li, Jingjing Dai, Xilei Cai, Xuejiao Ma, Yunzhu Zhu, Jiabin Li

**Affiliations:** 1 Department of Infectious Diseases, The First Affiliated Hospital of Anhui Medical University, Hefei 230022, China; 2445011595@stu.ahmu.edu.cn (X.L.); yfy1241449@fy.ahmu.edu.cn (X.M.); 2 Anhui Center for Surveillance of Bacterial Resistance, The First Affiliated Hospital of Anhui Medical University, Hefei 230022, China; 3 Anhui Province Key Laboratory of Infectious Diseases, Anhui Medical University, Hefei 230022, China; 4 Institute of Bacterial Resistance, Anhui Medical University, Hefei 230022, China; 5 Institute of Infectious Diseases, Anhui Medical University, Hefei 230022, China; 6 Clinical Medicine, First Clinical Medical College, Anhui Medical University, No.81, Meishan Road, Shushan District, Hefei 230032, China; 2545011644@stu.ahmu.edu.cn; 7 The Second Affiliated Hospital, Anhui Medical University, Hefei 230601, China; 2546010260@stu.ahmu.edu.cn

**Keywords:** sepsis, coagulation, platelet count, international normalized ratio, organ dysfunction

## Abstract

**Background**: Static coagulation measurements cannot distinguish transient fluctuation from sustained abnormality in sepsis. We assessed whether 0–24 h dynamic coagulation phenotypes preceded noncoagulation organ dysfunction during 24–72 h. **Methods**: We conducted a retrospective cohort study using the Medical Information Mart for Intensive Care IV (MIMIC-IV) for development and the eICU Collaborative Research Database (eICU-CRD) for component-level replication. Platelet count, international normalized ratio (INR) and partial thromboplastin time (PTT) were aligned to fixed 6 h grids. The two-marker coagulation deterioration index (CDI2) was the prespecified primary exposure; coagulation instability, recovery and cumulative coagulation burden (CCB) were prespecified secondary phenotypes. Modified Poisson models estimated adjusted risk ratios (aRRs) with 95% confidence intervals (CIs). Trajectories incorporating measurements after 24 h were excluded from temporally ordered association and prediction models. **Results**: Among 13,669 eligible MIMIC-IV sepsis stays, 2208 had complete five-organ outcome assessment; 1278 (57.9%) developed the composite. In the primary CDI2 analysis (*n* = 1002; 619 events), each MIMIC standard deviation was associated with the composite (aRR 1.051, 95% CI 1.007–1.098), but the strict treatment/device-truncated estimate attenuated (aRR 1.018, 95% CI 0.966–1.074). Prespecified secondary CCB2 was associated in observed care (*n* = 2185; aRR 1.076, 95% CI 1.033–1.122), remained associated after strict truncation (aRR 1.061, 95% CI 1.006–1.119), and was robust to measurement-intensity adjustment and selection weighting (aRR 1.089, 95% CI 1.009–1.175). CCB2 showed the largest associations with liver and renal worsening; these persisted after excluding cirrhosis and baseline liver dysfunction and were directionally reproduced for eICU kidney and liver components. **Conclusions**: The prespecified primary directional-deterioration signal was small and sensitive to treatment/device truncation. Prespecified secondary analyses consistently indicated that persistence of coagulation abnormality was more informative than short-term direction, particularly for hepatic and renal deterioration. These associative findings apply principally to patients with sufficient coagulation and organ monitoring and require prospective confirmation.

## 1. Background

Sepsis is defined by a dysregulated host response with life-threatening organ dysfunction, yet its course is heterogeneous and can change within hours [1,2,3,4,5,6,7,8]. Coagulation activation, endothelial injury, platelet consumption and impaired anticoagulant pathways are integral to this biology [9,10,11,12,13,14,15,16,17,18,19,20,21,22,23,24]. Conventional analyses usually summarize the first, nadir or peak platelet count, international normalized ratio (INR) or partial thromboplastin time (PTT). These summaries compress a time-evolving process into one value and cannot separate the magnitude of fluctuation, adverse or favorable direction, and persistence of abnormality.

Platelet and coagulation abnormalities are clinically relevant but mechanistically ambiguous. Platelet loss may reflect immunothrombosis, consumption, dilution, extracorporeal circuits, medication exposure, marrow response or combinations of these processes [25,26,27,28,29,30,31,32]. PTT may be prolonged by therapeutic unfractionated heparin (UFH), while blood products can produce apparent recovery that does not represent spontaneous resolution. Dynamic phenotyping may therefore provide information that static values miss, but its interpretation requires explicit time ordering, observation-process assessment and treatment-aware sensitivity analyses [33,34,35,36,37,38].

We operationalized four study-defined candidate dynamic coagulation phenotypes: the coagulation instability index (CII), coagulation deterioration index (CDI), coagulation recovery index (CRI) and cumulative coagulation burden (CCB). CII partitions exactly into CDI plus CRI; CCB quantifies persistence of ordinal abnormality across observed six-hour bins. These indices were newly defined for this study and are neither established scores nor clinically ready biomarkers. A five-class platelet-INR trajectory model spanning 0–72 h was retained only as a concurrent descriptive analysis because its last two windows overlap outcome ascertainment.

Kidney injury, sepsis-associated encephalopathy and liver dysfunction are well-recognized manifestations of sepsis [39,40,41,42,43,44,45,46]. We therefore focused on new or worsening noncoagulation organ dysfunction as a clinically proximal outcome, with the 0–24 h index exposure window strictly separated from the 24–72 h outcome window. Death was excluded from the primary organ-dysfunction endpoint to preserve its interpretation, and an organ-deterioration-or-death composite was examined as a sensitivity analysis.

Our primary objective was to test whether prespecified primary CDI2 during 0–24 h preceded a complete five-organ worsening composite during 24–72 h. Prespecified secondary objectives evaluated CII, CRI and CCB, treatment-aware versions, measurement and selection processes, and component-level cross-database consistency. We hypothesized that directional deterioration would be associated with the primary composite and that persistent abnormal burden might provide complementary information.

## 2. Methods

### 2.1. Study Design and Reporting

We performed a retrospective two-database cohort study. MIMIC-IV version 3.1 contains granular, time-stamped clinical data from Beth Israel Deaconess Medical Center during 2008–2022; its dense laboratory, intervention and hourly organ-support records permitted reconstructed Sepsis-3 onset, fixed-window exposure construction and temporally separated Sequential Organ Failure Assessment (SOFA) outcomes. eICU-CRD contains multicenter US intensive care unit (ICU) data from 2014 to 2015 and was selected for independent component-level replication across heterogeneous hospitals [47,48]. We followed the Strengthening the Reporting of Observational Studies in Epidemiology (STROBE) statement and used Transparent Reporting of a Multivariable Prediction Model for Individual Prognosis or Diagnosis (TRIPOD) principles for supporting prediction analyses [49,50]. Before organ-outcome modeling, we specified the primary exposure, primary outcome, covariates and treatment-aware sensitivities.

### 2.2. Population and Time Zero

Adults aged at least 18 years with a first ICU stay meeting the official MIMIC Sepsis-3 implementation were eligible. Reconstructed sepsis onset defined t0 and was required to occur from 24 h before to 24 h after ICU admission; the ICU stay had to last at least 24 h. Pregnancy-related admissions, direct cardiac-surgical or cardiopulmonary-bypass ICU admissions, acute coronary syndromes receiving urgent revascularization, myocarditis, aortic dissection, pulmonary embolism causing right-heart injury, pre-sepsis cardiogenic shock and pre-t0 extracorporeal membrane oxygenation (ECMO) were excluded. In eICU, ICU admission defined t0 because equivalent Sepsis-3 onset reconstruction was unavailable. A MIMIC sensitivity analysis re-anchored exposure and outcome windows at ICU admission.

### 2.3. Laboratory Harmonization and Fixed Grid

Platelet count, INR and PTT were harmonized by label, unit, reference range and clinically plausible limits. Duplicate specimen records were removed, and values within each fixed 6 h bin were summarized by the median. No last observation carried forward was used. Change indices used only adjacent observed bins separated by exactly 6 h; each included marker required at least two valid bins and one adjacent pair. Platelet abnormality was oriented downward, and INR/PTT abnormality upward. Development-set robust medians and 1.4826 × median absolute deviation scales were frozen and applied in eICU without refitting. For eICU, the PTT upper limit of normal was set at 36.5 s after harmonized reference-range review.

### 2.4. Dynamic Coagulation Measures

For marker m, adverse-oriented robust z-scores were calculated from untransformed platelet values and log-transformed INR/PTT values. Across adjacent observed bins, marker-specific CII, CDI and CRI were the means of |Δz|, max(Δz,0) and max(−Δz,0), respectively; each index was then averaged across the required markers, so CII = CDI + CRI. CCB was an observed-bin mean rather than an interpolated area. Platelet burden scores were 0/1/2/3 for ≥150/100–149/50–99/<50 × 10^9^/L, and INR scores were 0/1/2/3 for ≤1.2/1.21–1.4/1.41–1.8/>1.8. CCB2 was the mean platelet-plus-INR score across bins in which both were observed; unobserved bins were excluded, without interpolation or zero filling, and at least one joint bin was required. CCB3 additionally required and scored PTT. CDI2 was the prespecified primary exposure; CII2, CRI2, CCB2 and three-marker versions were prespecified secondary exposures. Full equations are provided in the Supplementary Methods.

### 2.5. Treatment-Aware Versions

The observed-care version used every valid measurement during the exposure window. The pre-direct-treatment version truncated at the earliest platelet, plasma, cryoprecipitate, prothrombin complex concentrate, vitamin K, protamine or therapeutic anticoagulant event. The strict version additionally truncated at the start of continuous renal replacement therapy (CRRT), ECMO or another major extracorporeal circuit. Prophylactic and therapeutic anticoagulation were not combined, and circuit/device heparin was represented separately. Red-cell transfusion remained a covariate but did not define direct coagulation treatment. A same-population sensitivity restricted observed-care analyses to patients eligible for the corresponding strict index.

### 2.6. Joint Trajectory Model

The five-class joint platelet-INR trajectory model used the 0–6, 6–12, 12–24, 24–48 and 48–72 h windows, plotted at midpoints 3, 9, 18, 36 and 60 h. Eligibility required at least one early and one 24–72 h value for both markers; the trajectory cohort contained 10,421 stays, and bootstrap validation yielded a median adjusted Rand index of 0.855. Because the 24–48 and 48–72 h values overlapped the organ-outcome window, trajectory classes were treated only as concurrent descriptive phenotypes and were excluded from all prospective association and prediction models (Supplementary Figure S9; Supplementary Table S15).

### 2.7. Outcomes

For each MIMIC Sequential Organ Failure Assessment (SOFA) respiratory, cardiovascular, central nervous system (CNS), liver and renal component, baseline was the maximum score during 0–24 h and outcome burden was the maximum during 24–72 h. An event required an outcome score of at least 2 and an increase of at least 1 point. The primary composite was any event among stays with all five components evaluable. Secondary outcomes included component events, at least two worsening organs, change in the sum of component maxima, Kidney Disease: Improving Global Outcomes (KDIGO) worsening, new invasive ventilation and new vasoactive support. Sensitivities summarized outcome evaluability by death, ICU discharge and continued ICU stay; combined organ deterioration with death; and accepted any observed worsening when at least three organ components were evaluable.

### 2.8. Severity-Gradient Sensitivity

Severe organ escalation required an outcome component score of at least 3 and an increase of at least 2 points. Severe multiorgan escalation required this rule in at least two organs. We also evaluated summed noncoagulation SOFA increases of at least 2 and at least 4 points. MIMIC-derived quartiles for CDI2 and CCB2 were applied unchanged in eICU. To avoid threshold-driven interpretation, we reported Q4-versus-Q1 estimates together with all category-specific event rates and continuous results.

### 2.9. Covariates and Missing Data

Candidate covariates included age, sex, race, comorbidity burden, infection source, baseline noncoagulation SOFA, lactate, mean arterial pressure, mechanical ventilation, vasoactive support, creatinine, bilirubin and relevant chronic diseases. Covariate counts were limited by prespecified event-per-parameter information gates. Exposures and outcomes were never imputed. Missing prespecified covariates were imputed in 20 chained-equation datasets with stochastic conditional-model draws and combined with Rubin’s rules [51]. Troponin (58.5% missing) and albumin (64.6% missing) were displayed descriptively but were excluded from all prespecified covariate tiers and imputation equations.

### 2.10. Statistical Analysis

Modified Poisson regression with robust standard errors estimated adjusted risk ratios and 95% CIs [52]. The continuous CDI2-primary-composite model was the prespecified primary analysis. CII2, CRI2, CCB2, three-marker versions, treatment-aware versions and organ components were secondary analyses; severity gradients, restricted cubic splines, Kaplan–Meier curves, Cox models, concurrent trajectories and subgroups were exploratory. No multiplicity adjustment was applied to secondary or exploratory analyses; their *p*-values were treated as descriptive, with emphasis on effect sizes, CIs, analytic consistency and external directional replication. Prespecified subgroups were age, sex, baseline noncoagulation SOFA, early vasoactive support, early ventilation, pulmonary source, cirrhosis and stage 4/5 chronic kidney disease; heterogeneity was assessed by interaction *p*-values.

### 2.11. Validation

Supporting internal prediction analyses used MIMIC anchor years 2008–2019 for development and the latest anchor-year group, 2020–2022, as the temporal holdout. Base, static, dynamic and full-without-trajectory models were compared by area under the receiver operating characteristic curve (AUC), precision-recall AUC, Brier score, calibration, bootstrap intervals and decision curves. The trajectory variable was excluded because it used measurements after 24 h. External analyses used eICU-compatible kidney and liver thresholds and documentation-based support outcomes. Because the complete five-organ SOFA composite could not be reconstructed reliably, we described these analyses as component-level replication and applied MIMIC-derived scales, coefficients and category boundaries unchanged. Hospital clustering and the number of contributing hospitals were reported where estimable.

### 2.12. Dose–Response and Event-Time Analyses

Organ-specific dose–response and event-time analyses were exploratory. Restricted cubic spline models evaluated observed-care CDI2 and CCB2 with four knots at the 5th, 35th, 65th and 95th percentiles and a three-knot fallback for duplicate point masses. Kaplan–Meier and adjusted Cox analyses began at the 24 h landmark; event-free stays were censored at the last observed component hour or 72 h. Efron’s method handled ties, covariates were multiply imputed and Schoenfeld-residual time-trend tests assessed proportional hazards. Because the respiratory CCB2 model violated proportional hazards, a piecewise time-varying sensitivity analysis estimated separate 24–48 h and 48–72 h effects. Event-time analyses remained descriptive because discharge-related censoring may be informative.

## 3. Results

### 3.1. Study Population and Measurement Coverage

**Figure 1:**
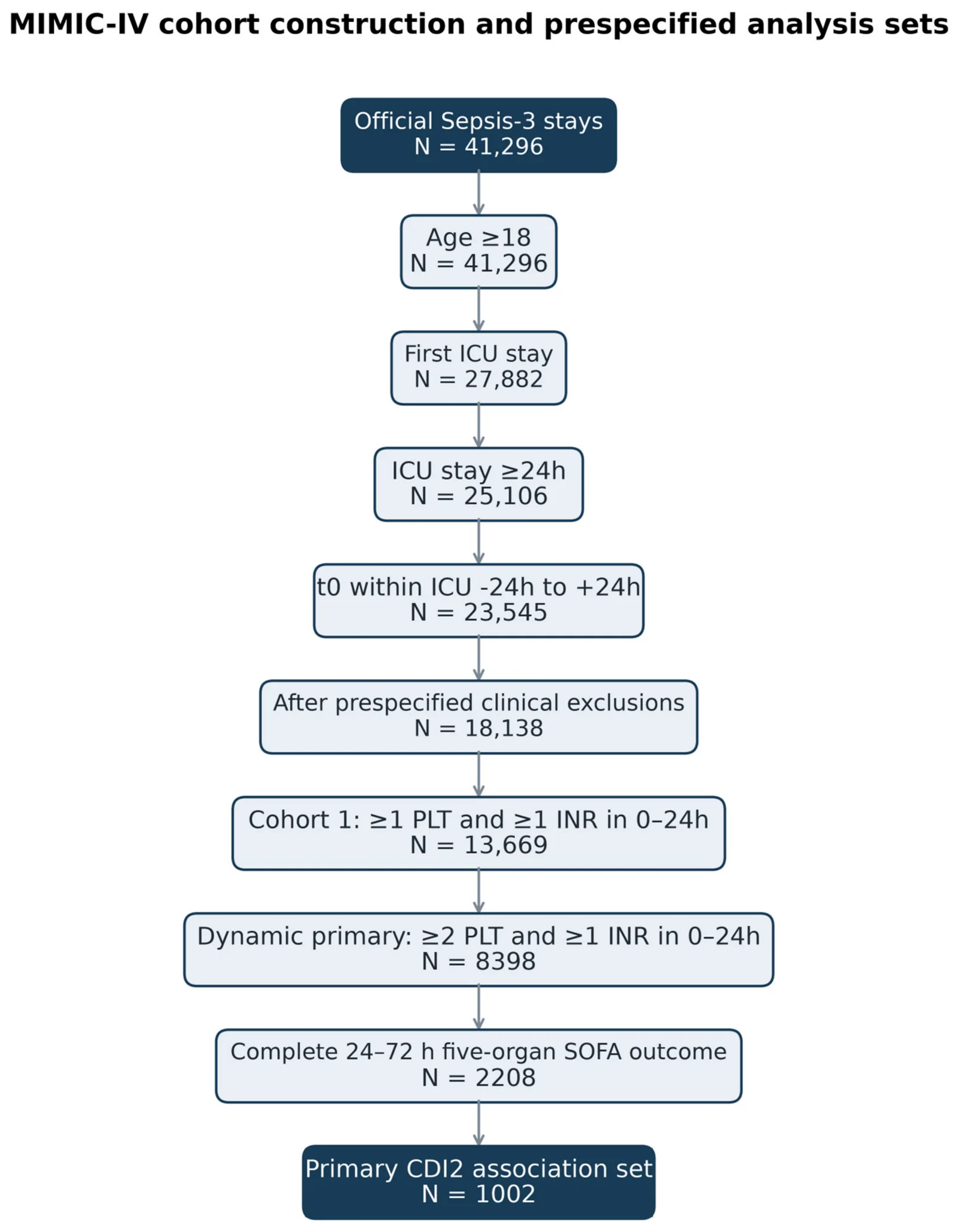
Cohort construction and analysis sets. MIMIC-IV cohort construction from the official Sepsis-3 population to the complete 24–72 h five-organ outcome set and primary CDI2 association set. Death was excluded from the primary composite and incorporated in a prespecified sensitivity analysis.

We first quantified cohort retention and measurement coverage. The MIMIC-IV flow yielded 13,669 eligible sepsis stays. Complete 24–72 h assessment of all five noncoagulation SOFA components was available for 2208 stays; 1278 (57.9%) met the primary composite. The primary CDI2 analysis included 1002 stays and 619 events; the secondary CCB2 analysis included 2185 stays and 1268 events. At 72 h, adjacent-pair coverage was 7065 stays for platelets, 4786 for INR and 5258 for PTT. eICU yielded 8335 stays for its partial component framework, with less complete INR and PTT coverage (Figure 1; Supplementary Tables S1, S2, S8 and S24).

### 3.2. Baseline Organ Injury and Outcome Profile

**Figure 2:**
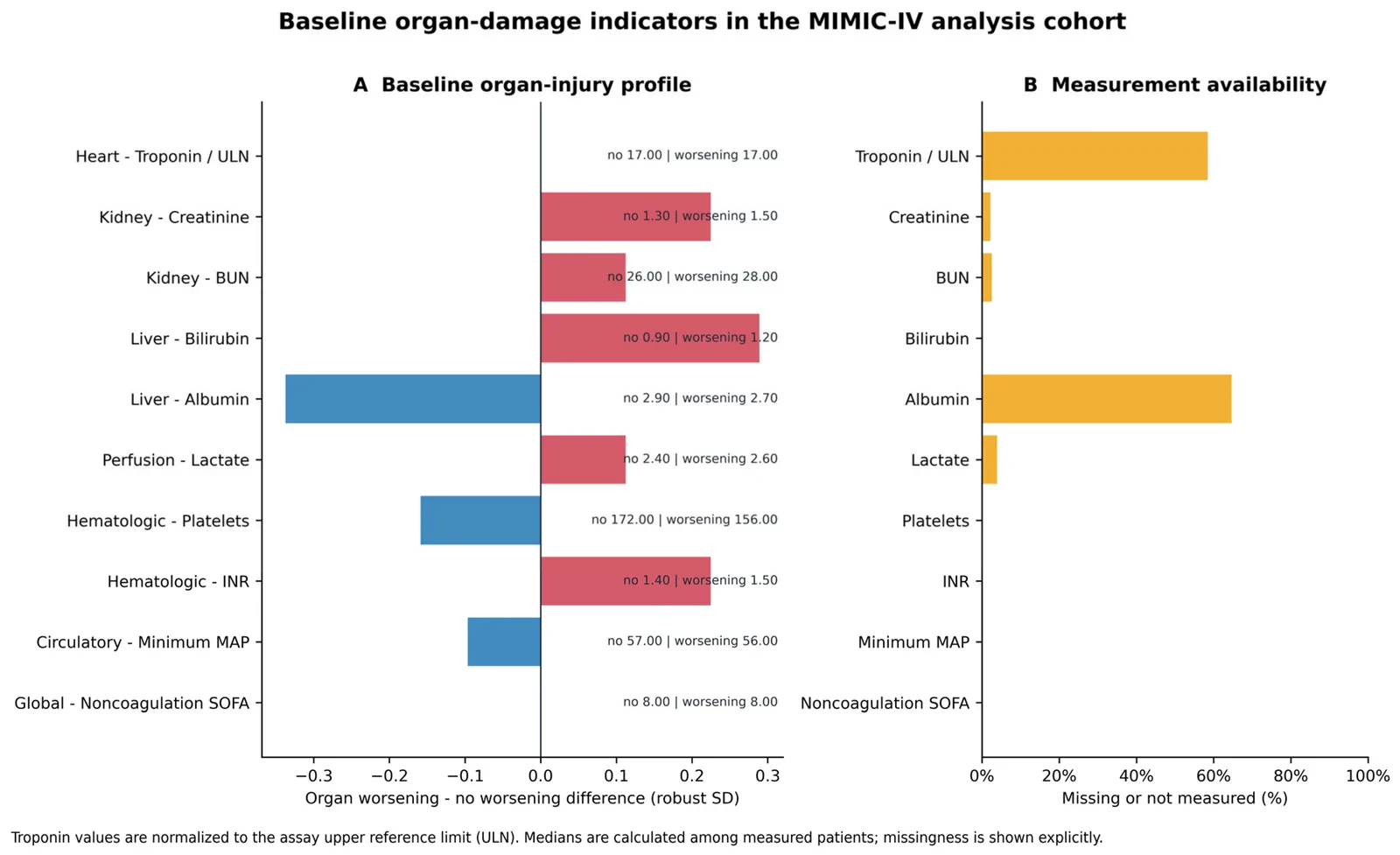
Baseline organ injury profiles. Baseline 0–24 h organ injury indicators among stays with complete five-organ outcome assessment, including troponin normalized to the assay upper limit of normal, creatinine, blood urea nitrogen, bilirubin, albumin, lactate, hemoglobin, platelet count, INR, minimum mean arterial pressure, ventilation, vasoactive support, and noncoagulation SOFA. Missingness is shown explicitly.

To describe baseline injury and clarify high-missingness variables, we retained the complete missingness display. Troponin was measured in 41.5% of the complete-outcome cohort (58.5% missing), and albumin was 64.6% missing. Both were descriptive only and were excluded from covariate tiers and chained-equation imputation. Creatinine, bilirubin, lactate, hemoglobin, platelet count, INR, minimum mean arterial pressure, ventilation, vasoactive support and noncoagulation SOFA were displayed with their observed availability (Figure 2; Supplementary Table S1).

### 3.3. Outcome Frequency and Severity

We next characterized the frequency and severity of each outcome before evaluating coagulation associations. Respiratory worsening was most frequent (915/4259; 21.5%), followed by CNS (1778/12,639; 14.1%), renal (1656/12,699; 13.0%), liver (504/5377; 9.4%) and cardiovascular worsening (652/12,942; 5.0%). Severe-grade events were most frequent for CNS dysfunction (7.49%) and renal dysfunction (6.15%), followed by respiratory (4.30%), cardiovascular (3.63%) and liver dysfunction (0.32%) (Supplementary Table S8).

### 3.4. Selection, Observation and Measurement-Process Analyses

We then assessed how the observation process shaped the principal analysis sets. Compared with all eligible stays, the complete-outcome cohort had higher first-day SOFA (median 10 vs. 6; standardized mean difference [SMD] 0.888), more mechanical ventilation (95.8% vs. 53.7%; SMD 1.110), more vasopressor use (68.1% vs. 40.6%; SMD 0.574), and more platelet and INR measurements. The CDI2 cohort was still more intensively sampled; hospital mortality was 38.8% vs. 21.9% in all eligible stays. Selection-weighted aRRs were 1.028 (95% CI 0.937–1.127) for CDI2 and 1.089 (95% CI 1.009–1.175) for CCB2. After adjustment for raw platelet/INR counts or valid six-hour bins, CCB2 aRRs were 1.060 and 1.063, respectively; among 1334 stays with at least two joint bins, the aRR was 1.077 (95% CI 1.022–1.135) (Supplementary Tables S24 and S25).

Among 13,535 patients alive at 24 h, 687 died during 24–72 h, 5162 were discharged alive before 72 h and 7686 remained in the ICU at 72 h; complete-composite evaluability was 25.0%, 1.9% and 25.2% in these states, respectively. When early death was combined with organ deterioration, aRRs were 1.052 (95% CI 1.023–1.082) for CDI2 and 1.061 (95% CI 1.031–1.092) for CCB2. With a less restrictive endpoint requiring at least three evaluable organs, estimates were 1.038 (95% CI 0.999–1.078) and 1.066 (95% CI 1.036–1.096), respectively (Supplementary Table S25).

### 3.5. Dynamic Measures and Concurrent Trajectories

**Figure 3:**
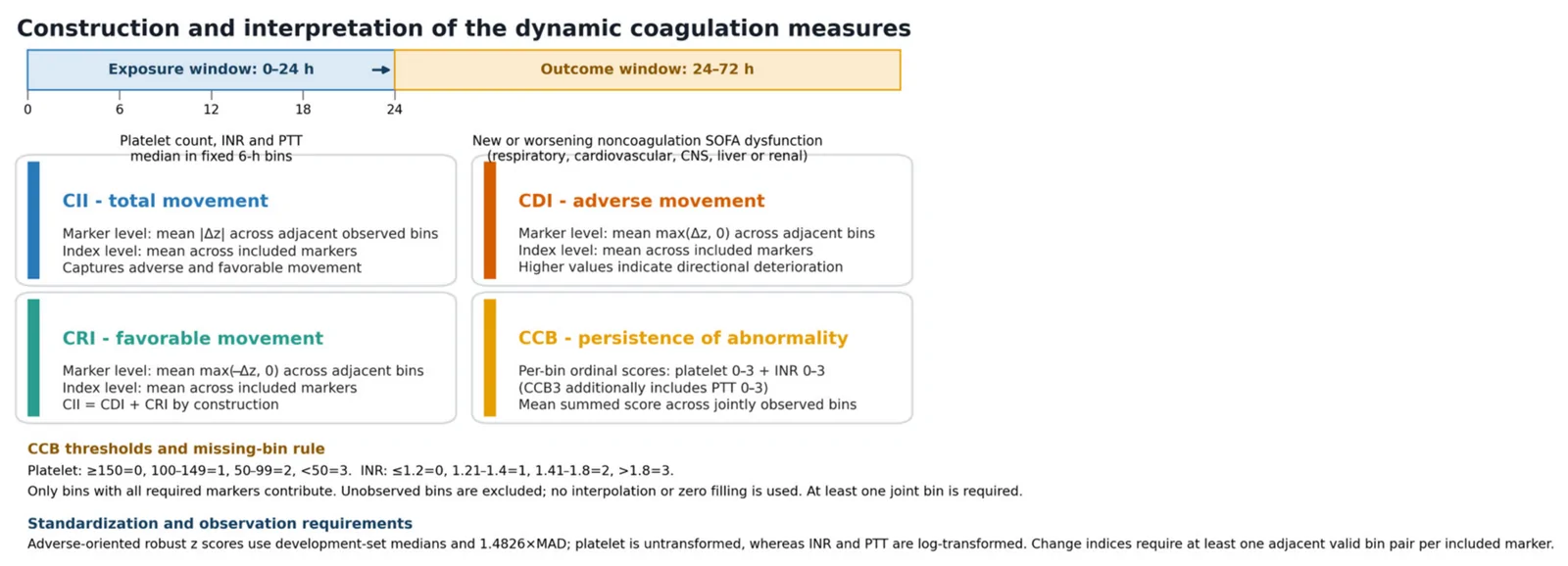
Construction and interpretation of the dynamic coagulation measures. The schematic distinguishes total movement (CII), adverse movement (CDI), favorable movement (CRI) and persistence of ordinal abnormality (CCB), together with exposure-outcome timing, observation requirements and missing-bin handling. Two-marker indices use platelet count and INR; three-marker indices additionally use PTT.

We then verified construct behavior before outcome modeling. All computational checks confirmed CII = CDI + CRI within numerical tolerance. CCB retained more observations than change indices because it required one joint observed bin rather than an adjacent pair. The five trajectory classes used measurements at 3, 9, 18, 36 and 60 h; the last two time points overlapped the outcome window. Accordingly, the trajectories are reported only as concurrent descriptive profiles, without organ-association estimates or inclusion in prediction models (Figure 3; Supplementary Figures S1 and S9; Supplementary Tables S2, S3 and S15).

### 3.6. Primary and Secondary Organ-Dysfunction Associations

**Figure 4:**
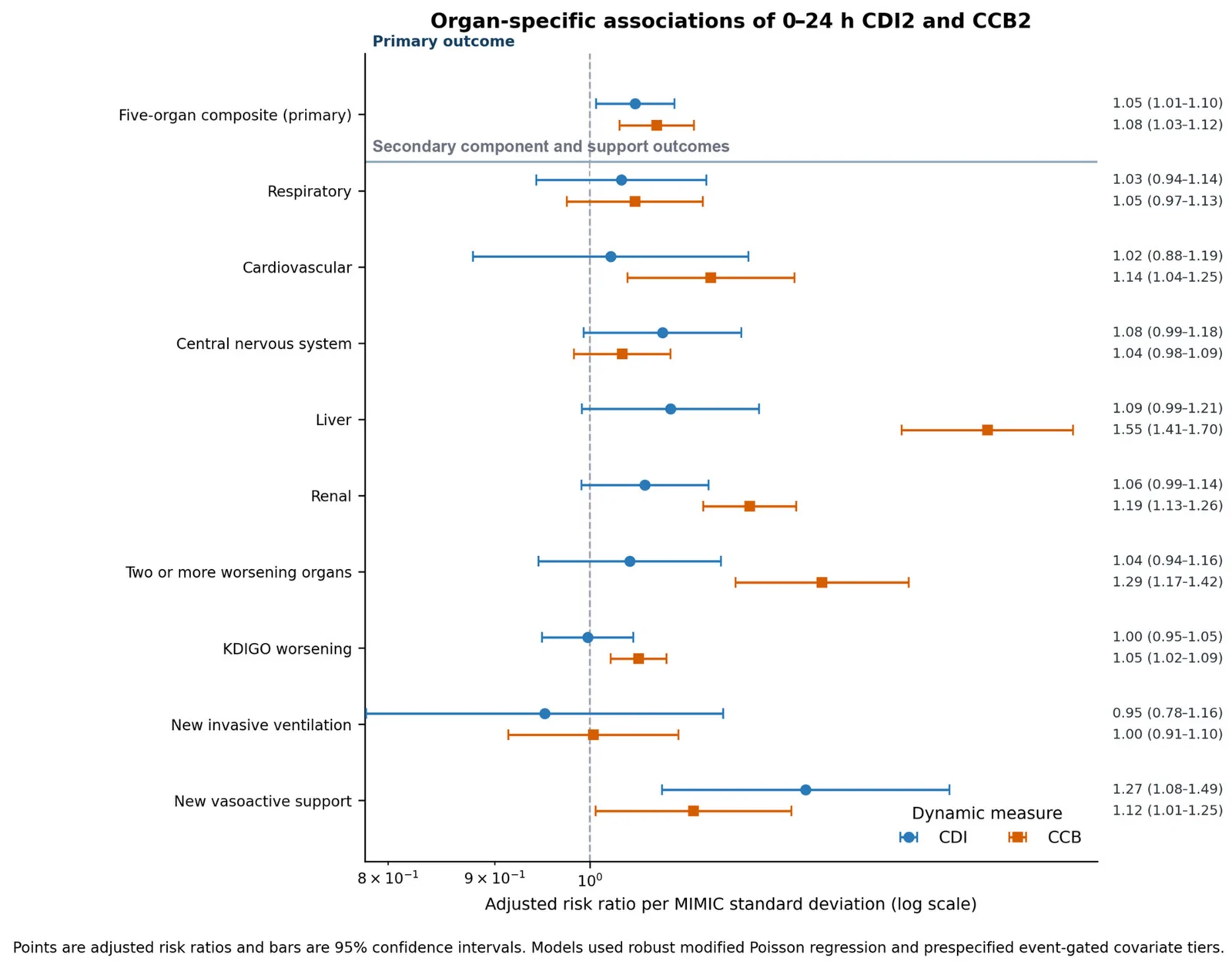
Primary composite and secondary organ-specific associations. Adjusted risk ratios and 95% CIs per MIMIC standard deviation of observed-care CDI2 and CCB2. The primary complete five-organ composite is visually separated from prespecified secondary components and support outcomes. Robust modified Poisson models used event-gated covariate tiers.

We next tested the prespecified primary exposure and prespecified secondary phenotypes. In observed care, each MIMIC standard deviation of primary CDI2 was associated with the complete five-organ composite (aRR 1.051, 95% CI 1.007–1.098). Secondary CII2 (aRR 1.069, 95% CI 1.023–1.117) and CCB2 (aRR 1.076, 95% CI 1.033–1.122) were also associated. CCB2 had the largest continuous associations with liver worsening (aRR 1.550, 95% CI 1.410–1.704) and renal worsening (aRR 1.193, 95% CI 1.133–1.255), whereas CDI2 was most strongly associated with new vasoactive support (aRR 1.269, 95% CI 1.083–1.487). Figure 4 visually separates the primary composite from secondary component and support outcomes (Figure 4; Supplementary Table S4).

### 3.7. Treatment-Timing Sensitivity

**Figure 5:**
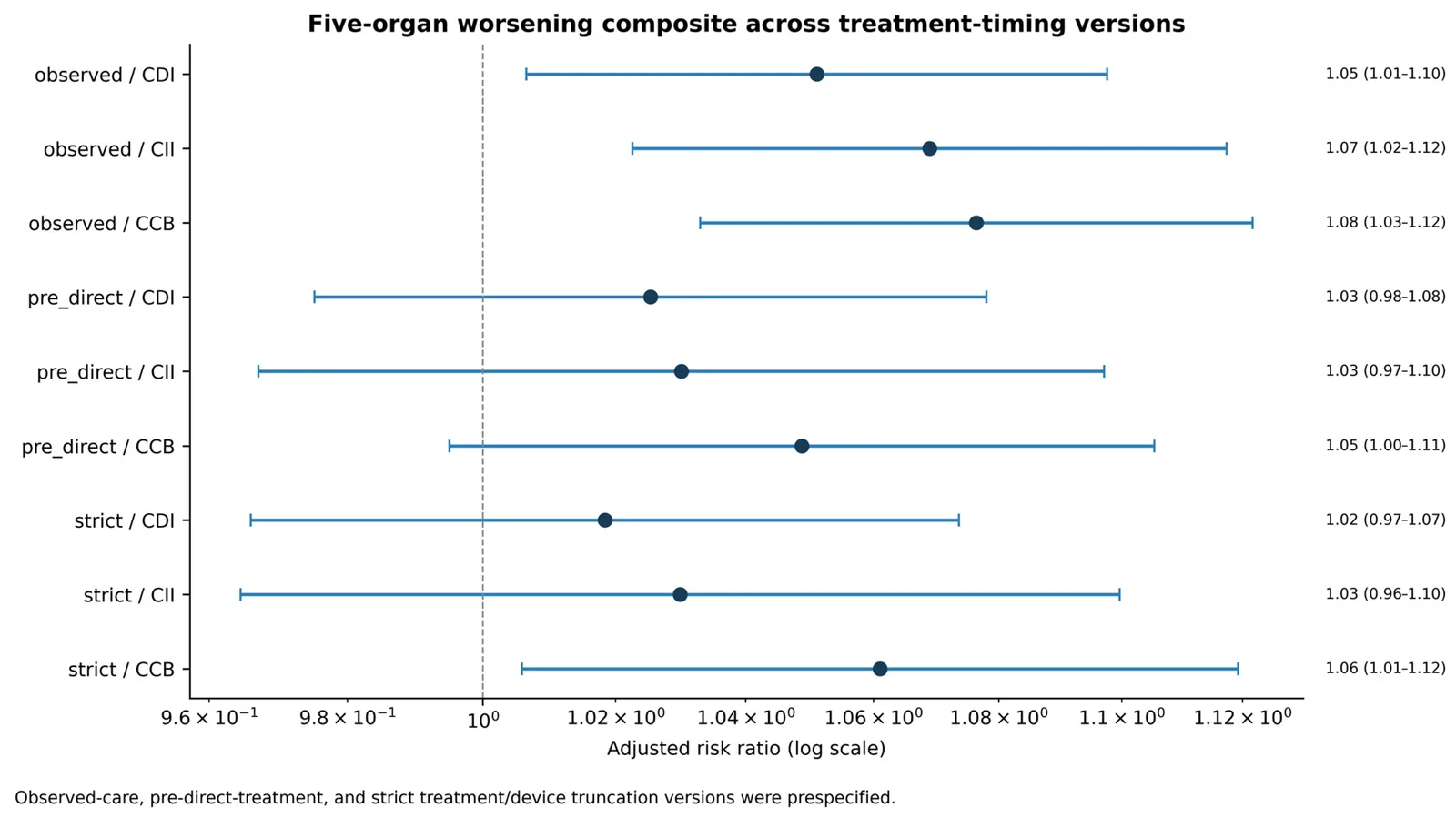
Treatment-timing sensitivity of composite associations. Adjusted associations of CDI2, CII2, and CCB2 with the five-organ composite across observed-care, pre-direct-treatment, and strict treatment/device-truncated versions. Different analysis sets result from the requirement for adequate pre-intervention measurements.

To assess treatment timing without conflating it with population change, we examined both conventional and same-population estimates. Pre-direct-treatment CDI2 and CII2 attenuated to aRRs of 1.025 (95% CI 0.975–1.078) and 1.030 (95% CI 0.967–1.097); under strict treatment/device truncation, their aRRs were 1.018 (95% CI 0.966–1.074) and 1.030 (95% CI 0.965–1.100). Secondary CCB2 was more stable (strict aRR 1.061, 95% CI 1.006–1.119). Among the identical strict-eligible population, observed-care and strict CCB2 estimates were 1.058 (95% CI 1.002–1.117) and 1.061 (95% CI 1.006–1.119), respectively; corresponding CDI2 estimates were 1.024 and 1.018 with overlapping CIs. This pattern indicates that both treatment-linked measurement changes and eligibility affect change indices, while the CCB2 estimate was less sensitive to truncation (Figure 5; Supplementary Tables S4, S5 and S25).

### 3.8. Targeted Liver and Time-Origin Sensitivities

We next tested whether the hepatic gradient reflected established liver disease and whether database time origins explained cross-database consistency. The CCB2-liver association persisted after excluding cirrhosis (aRR 1.500, 95% CI 1.305–1.725), restricting baseline liver SOFA to <2 (aRR 1.419, 95% CI 1.243–1.620), and applying both restrictions (aRR 1.430, 95% CI 1.204–1.697). Re-anchoring MIMIC at ICU admission, which preceded reconstructed sepsis onset by a median 1.52 h, yielded CCB2 aRRs of 1.097 (95% CI 1.054–1.142) for the complete composite, 1.539 (95% CI 1.399–1.694) for liver worsening and 1.206 (95% CI 1.145–1.269) for renal worsening (Supplementary Table S25).

### 3.9. Organ-Specific Severity Gradients

**Figure 6:**
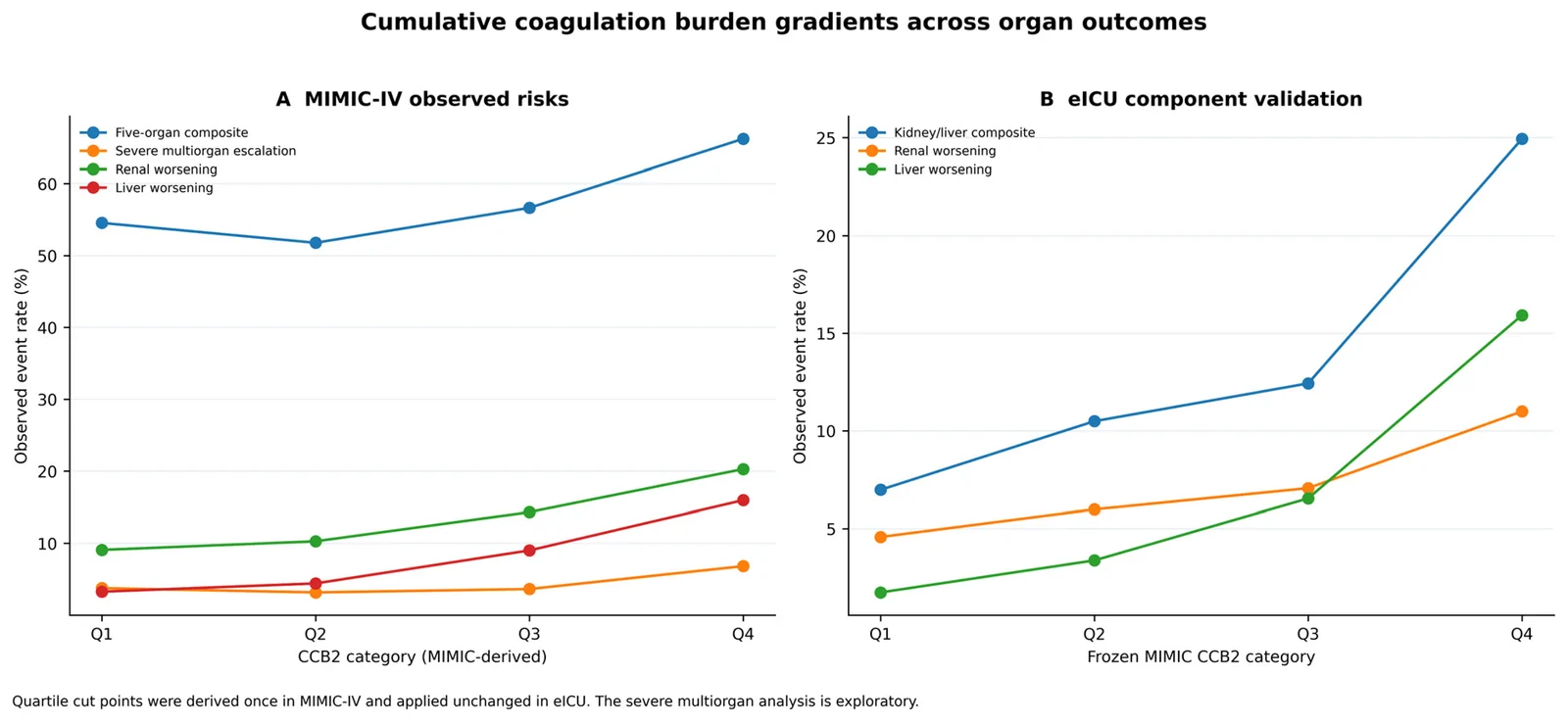
Coagulation burden gradients across organ outcomes. Observed risks across CCB2 categories in MIMIC-IV and eICU. Category boundaries were derived once in MIMIC-IV and applied unchanged in eICU. The severe multiorgan outcome was evaluated as an exploratory sensitivity analysis.

Observed risks rose across CCB2 categories, most clearly for liver, renal, and severe multiorgan outcomes. In MIMIC-IV, adjusted CCB2 Q4-versus-Q1 RRs were 1.175 (95% CI 1.031–1.340) for the primary composite, 1.656 (95% CI 1.417–1.936) for renal worsening, and 3.977 (95% CI 2.571–6.154) for liver worsening. Severe multiorgan escalation occurred in 98 model events and had an RR of 3.158 (95% CI 1.731–5.762) using the minimal covariate tier. Its raw Q1 and Q4 risks were 3.76% and 6.82%, respectively; the large relative estimate therefore represents a low-frequency exploratory contrast and should be interpreted as hypothesis-generating (Figure 6; Supplementary Figure S5; Supplementary Tables S11 and S12).

### 3.10. Dose–Response Relationships

**Figure 7:**
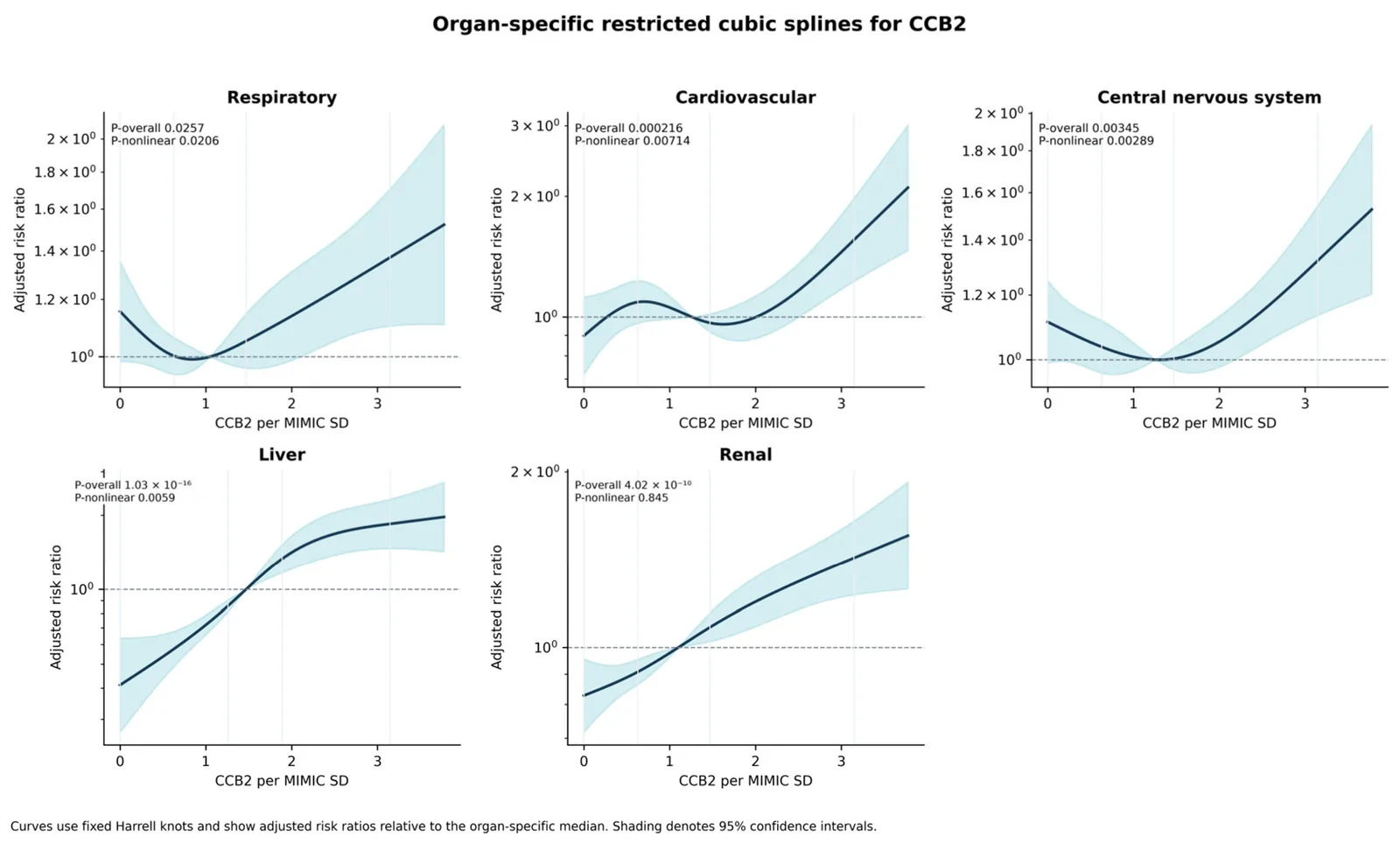
Organ-specific CCB2 dose–response curves. Adjusted restricted cubic spline curves for observed-care CCB2 and each organ-specific outcome. Four fixed Harrell knots were used, with the organ-specific median as reference. Overall and nonlinear Wald *p*-values are displayed. Shading denotes 95% confidence intervals.

CCB2 showed an overall spline association with every organ outcome. Evidence of nonlinearity was present for respiratory (*p* = 0.021), cardiovascular (*p* = 0.007), central nervous system (*p* = 0.003), and liver worsening (*p* = 0.006), whereas the renal curve was compatible with a predominantly linear increase (*p* for nonlinearity = 0.845). None of the five adjusted CDI2 spline tests showed an overall association (Figure 7; Supplementary Figure S6; Supplementary Tables S16 and S17).

### 3.11. Kaplan–Meier and Log-Rank Analyses

**Figure 8:**
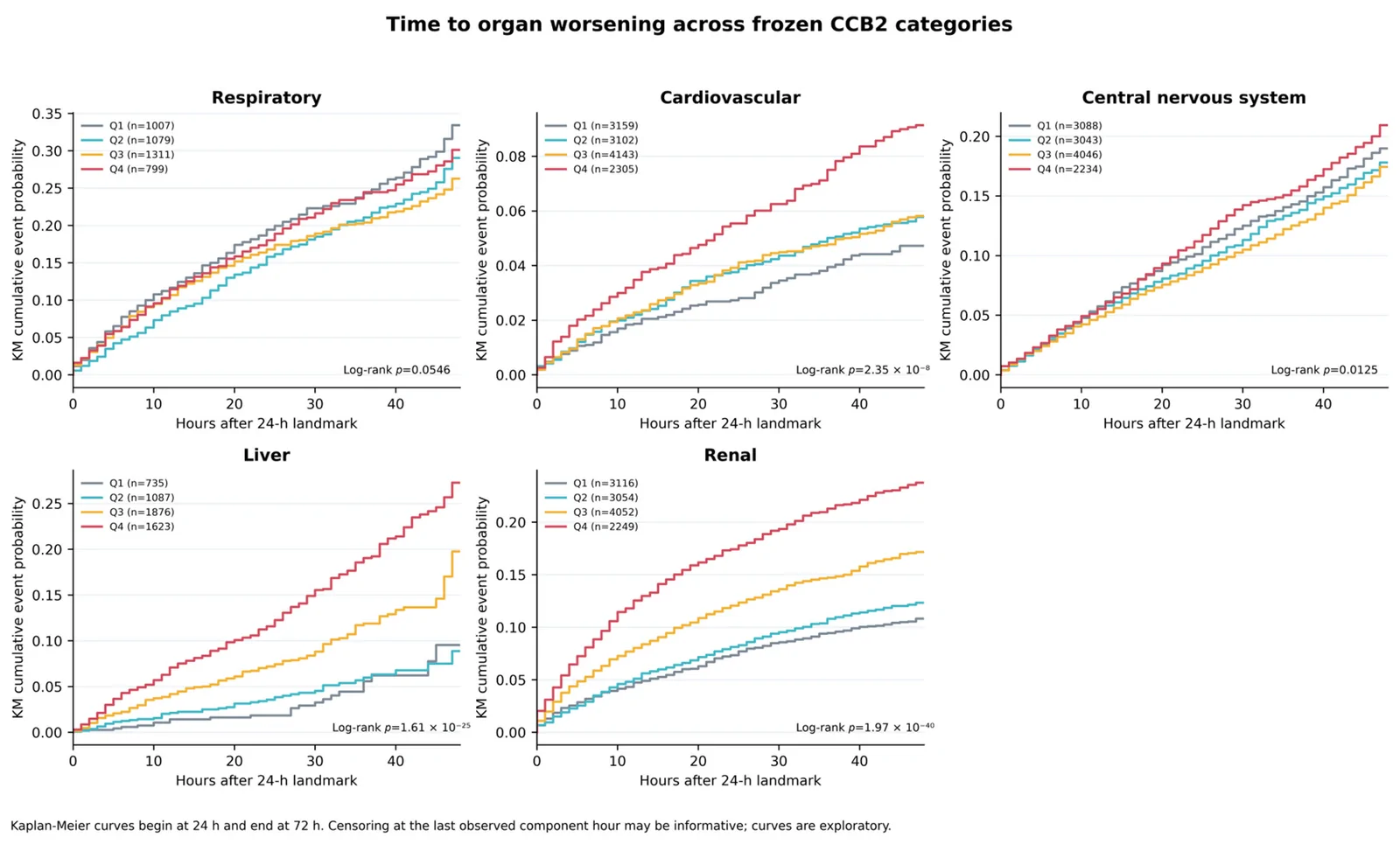
Organ worsening across CCB2 categories. Kaplan–Meier-derived cumulative event curves from the 24 h landmark through 72 h across MIMIC-derived CCB2 categories. Multigroup log-rank *p*-values compare all four categories. Event-free stays were censored at the last observed organ-component hour or 72 h; censoring may be informative.

Kaplan–Meier curves across MIMIC-derived CCB2 categories differed for cardiovascular, central nervous system, liver, and renal worsening (all log-rank *p* ≤ 0.013), while respiratory separation was borderline (*p* = 0.055). CDI2 category curves differed for liver and renal event timing but not for the other organs; because these log-rank comparisons were unadjusted and the corresponding CDI2 spline models were null, they were treated as descriptive rather than independent confirmation (Figure 8; Supplementary Figure S7; Supplementary Tables S18–S20).

### 3.12. Event-Time Cox Models

The event-time models were used to examine when recorded organ worsening occurred. Continuous CCB2 hazard ratios (HRs) were largest for liver (HR 1.554, 95% CI 1.402–1.722), followed by renal (HR 1.218, 95% CI 1.151–1.289) and cardiovascular worsening (HR 1.154, 95% CI 1.050–1.268); continuous CDI2 estimates were imprecise. The respiratory CCB2 proportional-hazards test was significant (*p* = 0.011), so a single constant HR was not retained. In the piecewise sensitivity analysis, the HR was 1.131 (95% CI 1.026–1.246) during 24–48 h and 1.013 (95% CI 0.881–1.165) during 48–72 h; the interval interaction *p*-value was 0.150. Thus, the respiratory association appeared concentrated earlier, although evidence for a difference between intervals was not conclusive (Supplementary Figure S8; Supplementary Tables S21–S23 and S25).

### 3.13. Subgroup Analyses and Internal Temporal Validation

We evaluated heterogeneity and supporting prediction performance after removing the overlapping trajectory variable. Most prespecified interaction tests were not significant; the isolated CDI2-by-sex interaction (*p* = 0.025) was interpreted cautiously without multiplicity adjustment. The dynamic model yielded an AUC of 0.602 in the 2020–2022 holdout, compared with 0.575 for the base model. The full model without trajectory had an AUC of 0.604 (bootstrap 95% interval 0.509–0.692), indicating modest and uncertain discrimination. These findings support association and phenotyping rather than standalone clinical prediction (Supplementary Figures S2–S4; Supplementary Tables S6, S9 and S10).

### 3.14. External Component-Level Validation

**Figure 9:**
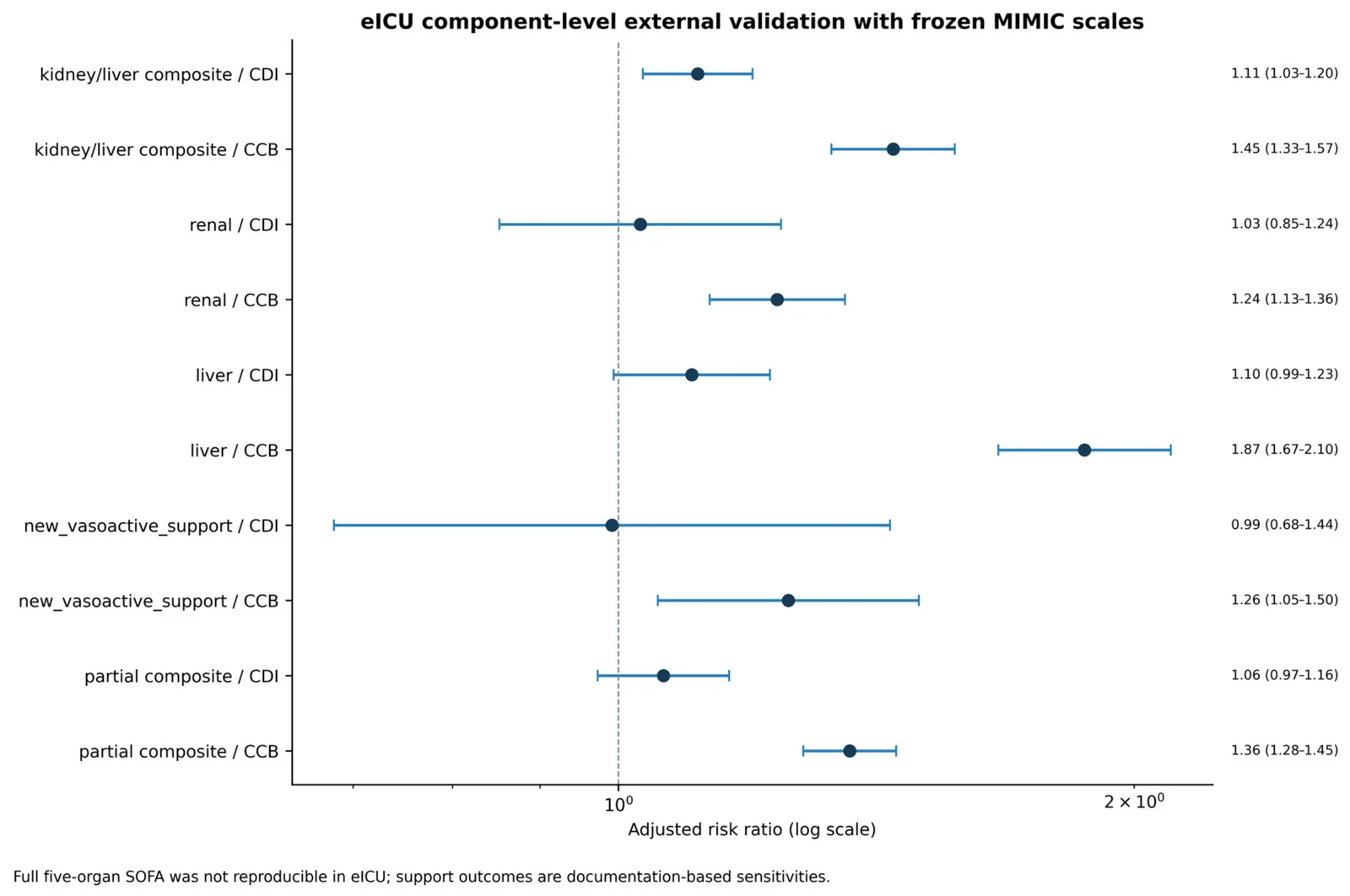
Component-level external validation in eICU. Adjusted risk ratios and 95% confidence intervals for eICU-compatible kidney, liver, partial-composite, and new vasoactive-support outcomes using MIMIC scales applied unchanged. The complete five-organ SOFA outcome was not reproducible under the prespecified eICU completeness requirements.

Finally, we assessed component-level cross-database consistency using unchanged MIMIC parameters. In eICU, the kidney/liver laboratory composite occurred in 559/3878 evaluable stays (14.4%), renal worsening in 549/7725 (7.1%), liver worsening in 293/3893 (7.5%) and new vasoactive support in 216/8335. On the MIMIC scale, continuous CCB2 aRRs were 1.447 (95% CI 1.332–1.572) for the kidney/liver composite, 1.238 (95% CI 1.130–1.356) for renal worsening and 1.871 (95% CI 1.667–2.101) for liver worsening. CCB2 analyses drew from 168 hospitals for the kidney/liver and liver outcomes, 183 for renal worsening and 186 for support outcomes; the smaller CDI2/CII2 sets drew from 97 to 110 hospitals. Because the full five-organ endpoint was unavailable and eICU used ICU admission rather than reconstructed sepsis onset, these results represent component-level directional replication rather than validation of the overall construct (Figure 9; Supplementary Tables S7, S13, S14 and S25).

## 4. Discussion

This study distinguished a prespecified primary directional-change hypothesis from prespecified secondary burden analyses. Primary CDI2 showed a small association with the complete five-organ composite in observed care and attenuated after strict treatment/device truncation. Secondary CCB2 was more stable across timing, selection, measurement-intensity and outcome-definition sensitivities and showed the largest liver and renal associations. The evidence therefore supports persistent coagulation abnormality as a candidate dynamic phenotype, while the marked selection into intensively monitored analysis sets limits population-wide generalization.

Our findings extend, rather than replace, prior longitudinal coagulation studies. Dhainaut and colleagues reported that first-day changes in prothrombin time, D-dimer and antithrombin were associated with progression to multiorgan failure and mortality [53]. A prospective study found that persistently high sepsis-induced coagulopathy scores over four days tracked 28-day mortality [54], and multicenter database studies linked platelet trajectories and delayed platelet recovery to mortality [55,56]. These studies agree that serial behavior matters, but they generally used mortality or concurrent organ-severity outcomes over several days. Our 0–24 h indices precede a separate 24–72 h organ window, exclude noncoagulation SOFA from the exposure and explicitly account for treatment timing; the overlapping 0–72 h trajectory was removed from prospective analyses.

The conceptual separation among the indices helps interpret the pattern. CII measures movement in either direction, while CDI and CRI partition adverse and favorable motion. CCB instead averages ordinal abnormality across jointly observed bins. In treatment-exposed ICU data, a transient platelet or INR change can reflect dilution, blood products, anticoagulation, device exposure or sampling noise. Persistent burden may integrate continued endothelial injury, immunothrombosis, platelet consumption, hepatic synthetic dysfunction and global severity [4,9,12,13,14,28]. This mechanistic compatibility does not establish that coagulation abnormalities cause later organ dysfunction.

Observation processes were consequential. The complete-outcome and CDI2 cohorts were younger but substantially sicker, more frequently ventilated and vasopressor-treated, and more intensively tested than all eligible stays. Selection-weighted CDI2 retained an above-unity point estimate but a wide CI, whereas CCB2 remained associated. CCB2 also persisted after adjustment for platelet/INR measurement counts or valid bins and was strongest among patients with at least two joint bins. These results reduce, but do not eliminate, surveillance-burden and collider-bias concerns; the principal estimand remains conditional on sufficient monitoring.

Treatment-aware truncation also changes both measurements and eligibility. The same-population analysis showed closely aligned observed and strict CCB2 estimates, while CDI2 remained imprecise in the smaller strict-eligible subset. Thus, the attenuation of directional indices cannot be attributed solely to removal of post-treatment values. The data more narrowly indicate that CCB2 was less sensitive to the implemented truncation and observation requirements than CDI2 and CII2.

The organ-specific pattern was internally coherent but requires calibrated interpretation. Respiratory worsening was most frequent, whereas liver worsening had the largest relative association with CCB2. The liver result persisted after excluding cirrhosis (aRR 1.500), restricting baseline liver SOFA to <2 (aRR 1.419), and applying both restrictions (aRR 1.430). This argues against pre-existing cirrhosis or established bilirubin-based dysfunction as the sole explanation, although INR may still reflect early hepatic synthetic failure, vitamin K status, anticoagulation or transfusion. Renal associations were smaller but consistent across fixed-window, severity-gradient and event-time analyses.

Cross-database consistency was deliberately assessed at the component level. eICU reproduced kidney and liver gradients with frozen MIMIC scales across many hospitals, but it lacked the timed respiratory and CNS information required for the primary composite. Its ICU-admission t0 also differs from MIMIC’s reconstructed sepsis onset. In MIMIC, sepsis onset occurred a median 1.52 h after ICU admission, and ICU-admission re-anchoring retained CCB2 associations with the composite (aRR 1.097), liver (aRR 1.539) and renal worsening (aRR 1.206). This supports robustness to the observed time-origin shift without making the two database windows biologically identical.

Dose–response and event-time analyses provided complementary descriptive evidence. CCB2 splines supported organ-specific gradients, whereas CDI2 spline tests were broadly null. Kaplan–Meier separation remains vulnerable to informative discharge and should not be read as biological latency. The respiratory proportional-hazards violation further demonstrates why a follow-up-wide HR can obscure change: the piecewise estimate was higher during 24–48 h than 48–72 h, although the formal interaction was not significant. Fixed-window risk ratios therefore remain the primary effect measure.

The trajectory analysis now has a strictly descriptive role. Its 36- and 60 h points are concurrent with outcome ascertainment and cannot be prospective predictors of 24–72 h deterioration. Presenting the profiles in the supplement preserves their phenotypic information while preventing future measurements from entering the association or prediction models. The resulting full temporal model, which excludes trajectory, has an AUC of 0.604 with a broad bootstrap interval and is supporting evidence only.

The study is consequently stronger as an association and phenotyping analysis than as a prediction study. AUCs near 0.60 are insufficient for standalone bedside prediction, autonomous alerting, or treatment selection. We therefore use the term candidate dynamic coagulation phenotype rather than digital biomarker or clinical score and do not propose replacement of SOFA or established coagulation assessments.

Strengths include explicit exposure-outcome separation, reproducible index equations, fixed grids without interpolation, treatment-aware and same-population analyses, covariate-only multiple imputation, selection and measurement-intensity sensitivities, transparent competing-state evaluability, targeted liver and time-zero analyses, and frozen component-level eICU replication. CDI2 remained the sole prespecified primary exposure. No multiplicity correction was applied to secondary and exploratory analyses; accordingly, inference rests on effect sizes, CIs and consistency rather than isolated *p*-values.

Limitations include retrospective design, residual confounding, informative testing, incomplete organ measurement, exposure-dependent eligibility and possible selection/collider bias. Inverse selection weighting depends on correctly measured predictors and cannot recover unmeasured reasons for testing or continued observation. Early death and discharge affect outcome evaluability; the organ-or-death and less restrictive analyses address this only partially. Baseline maxima may misclassify rapidly evolving dysfunction; CNS SOFA is sensitive to sedation; bilirubin may reflect chronic liver disease; and creatinine is affected by fluid balance and renal replacement therapy. eICU provided only component-level replication, and all secondary organ, categorical, spline, event-time and subgroup analyses remain exploratory and associative.

Prospective studies should enroll patients independently of laboratory frequency, standardize sampling, preserve the 0–24 h/24–72 h temporal split and validate CDI2 as the primary hypothesis while independently confirming CCB2. Direct platelet function, thrombin generation, fibrinolysis, endothelial and inflammatory markers could test whether persistent routine-test burden reflects a coherent biological state. Future models should incorporate time-varying treatment and observation processes and prioritize prespecified liver and kidney outcomes with absolute-risk reporting.

## 5. Conclusions

The prespecified primary CDI2 association was small and sensitive to treatment/device truncation. Prespecified secondary analyses consistently showed that persistent coagulation abnormality, represented by CCB2, was associated with subsequent noncoagulation organ dysfunction and was less sensitive to timing, selection and measurement-intensity analyses. Hepatic and renal gradients were directionally reproduced at the component level in eICU, but the full five-organ construct was not externally validated. These candidate phenotypes are associative, apply principally to sufficiently monitored patients and require prospective confirmation before clinical use.

## Data Availability

The de-identified MIMIC-IV and eICU-CRD datasets are available to credentialed users through PhysioNet under their respective data use agreements. Aggregate analysis outputs and reproducibility code contain no patient-level data.

## Supplementary Materials

The following supporting information can be downloaded at: https://www.mdpi.com/article/10.3390/jcm15218326/s1, supplementary figures (Figures S1–S9); supplementary tables (Tables S1–S25), supplementary methods.

## References

[1] Singer M., Deutschman C.S., Seymour C.W., Shankar-Hari M., Annane D., Bauer M., Bellomo R., Bernard G.R., Chiche J.-D., Coopersmith C.M. (2016). The Third International Consensus Definitions for Sepsis and Septic Shock (Sepsis-3). JAMA.

[2] Ranzani O.T., Singer M., Salluh J.I.F., Shankar-Hari M., Pilcher D., Berger-Estilita J., Coopersmith C.M., Juffermans N.P., Laffey J., Reinikainen M. (2025). Development and Validation of the Sequential Organ Failure Assessment (SOFA)-2 Score. JAMA.

[3] Seymour C.W., Liu V.X., Iwashyna T.J., Brunkhorst F.M., Rea T.D., Scherag A., Rubenfeld G., Kahn J.M., Shankar-Hari M., Singer M. (2016). Assessment of Clinical Criteria for Sepsis: For the Third International Consensus Definitions for Sepsis and Septic Shock (Sepsis-3). JAMA.

[4] Iba T., Levi M., Levy J.H. (2020). Sepsis-Induced Coagulopathy and Disseminated Intravascular Coagulation. Semin. Thromb. Hemost..

[5] Iba T., Helms J., Levy J.H. (2024). Sepsis-induced coagulopathy (SIC) in the management of sepsis. Ann. Intensive Care.

[6] Vincent J.L., Moreno R., Takala J., Willatts S., De Mendonça A., Bruining H., Reinhart C.K., Suter P.M., Thijs L.G. (1996). The SOFA (Sepsis-related Organ Failure Assessment) score to describe organ dysfunction/failure. On behalf of the Working Group on Sepsis-Related Problems of the European Society of Intensive Care Medicine. Intensive Care Med..

[7] Williams B., Zou L., Pittet J.F., Chao W. (2024). Sepsis-Induced Coagulopathy: A Comprehensive Narrative Review of Pathophysiology, Clinical Presentation, Diagnosis, and Management Strategies. Anesth. Analg..

[8] Shankar-Hari M., Phillips G.S., Levy M.L., Seymour C.W., Liu V.X., Deutschman C.S., Angus D.C., Rubenfeld G.D., Singer M. (2016). For the Sepsis Definitions Task Force. Developing a New Definition and Assessing New Clinical Criteria for Septic Shock: For the Third International Consensus Definitions for Sepsis and Septic Shock (Sepsis-3). JAMA.

[9] Iba T., Levy J.H., Warkentin T.E., Thachil J., van der Poll T., Levi M. (2019). The Scientific and Standardization Committee on DIC, and the Scientific and Standardization Committee on Perioperative and Critical Care of the International Society on Thrombosis and Haemostasis. Diagnosis and management of sepsis-induced coagulopathy and disseminated intravascular coagulation. J. Thromb. Haemost..

[10] Iba T., Helms J., Connors J.M., Levy J.H. (2023). The pathophysiology, diagnosis, and management of sepsis-associated disseminated intravascular coagulation. J. Intensive Care.

[11] Iba T., Helms J., Neal M.D., Levy J.H. (2023). Mechanisms and management of the coagulopathy of trauma and sepsis: Trauma-induced coagulopathy, sepsis-induced coagulopathy, and disseminated intravascular coagulation. J. Thromb. Haemost..

[12] Maneta E., Aivalioti E., Tual-Chalot S., Emini Veseli B., Gatsiou A., Stamatelopoulos K., Stellos K. (2023). Endothelial dysfunction and immunothrombosis in sepsis. Front. Immunol..

[13] Giustozzi M., Ehrlinder H., Bongiovanni D., Borovac J.A., Guerreiro R.A., Gąsecka A., Papakonstantinou P.E., Parker W.A. (2021). Coagulopathy and sepsis: Pathophysiology, clinical manifestations and treatment. Blood Rev..

[14] Tsantes A.G., Parastatidou S., Tsantes E.A., Bonova E., Tsante K.A., Mantzios P.G., Vaiopoulos A.G., Tsalas S., Konstantinidi A., Houhoula D. (2023). Sepsis-Induced Coagulopathy: An Update on Pathophysiology, Biomarkers, and Current Guidelines. Life.

[15] Aklilu A., Lai M.S., Jiang Z., Yip S.P., Huang C.L. (2025). Immunothrombosis in Sepsis: Cellular Crosstalk, Molecular Triggers, and Therapeutic Opportunities—A Review. In.t J. Mol. Sci..

[16] Li Y., Li H., Wang Y., Guo J., Zhang D. (2023). Potential Biomarkers for Early Diagnosis, Evaluation, and Prognosis of Sepsis-Induced Coagulopathy. Clin. Appl. Thromb. Hemost..

[17] Curtiaud A., Iba T., Angles-Cano E., Meziani F., Helms J. (2025). Biomarkers of sepsis-induced coagulopathy: Diagnostic insights and potential therapeutic implications. Ann. Intensive Care.

[18] Dempfle C.E. (2005). [Sepsis-associated coagulation disorders]. Hamostaseologie.

[19] Jacobi J. (2022). The pathophysiology of sepsis-2021 update: Part 1, immunology and coagulopathy leading to endothelial injury. Am. J. Health Syst. Pharm..

[20] Okamoto K., Tamura T., Sawatsubashi Y. (2016). Sepsis and disseminated intravascular coagulation. J. Intensive Care.

[21] Iba T., Umemura Y., Watanabe E., Wada T., Hayashida K., Kushimoto S. (2019). The Japanese Surviving Sepsis Campaign Guideline Working Group for Disseminated Intravascular Coagulation. Diagnosis of sepsis-induced disseminated intravascular coagulation and coagulopathy. Acute Med. Surg..

[22] Iba T., Levi M., Thachil J., Helms J., Scarlatescu E., Levy J.H. (2023). Communication from the Scientific and Standardization Committee of the International Society on Thrombosis and Haemostasis on sepsis-induced coagulopathy in the management of sepsis. J. Thromb. Haemost..

[23] Levi M., Schultz M., van der Poll T. (2013). Sepsis and thrombosis. Semin. Thromb. Hemost..

[24] Iba T., Nisio M.D., Levy J.H., Kitamura N., Thachil J. (2017). New criteria for sepsis-induced coagulopathy (SIC) following the revised sepsis definition: A retrospective analysis of a nationwide survey. BMJ Open.

[25] Li H., Zhou Y., Zhang X., Yao R., Li N. (2025). The relationship between hemoglobin, albumin, lymphocyte, and platelet (HALP) score and 28-day mortality in patients with sepsis: A retrospective analysis of the MIMIC-IV database. BMC Infect. Dis..

[26] Jiang H., Chen S., Gui X., Li Y., Sun Y., Zhu H., Dai Y., Zhang J., Li X., Ju W. (2025). Platelet NLRP6 protects against microvascular thrombosis in sepsis. Blood.

[27] Su M., Chen C., Li S., Li M., Zeng Z., Zhang Y., Xia L., Li X., Zheng D., Lin Q. (2022). Gasdermin D-dependent platelet pyroptosis exacerbates NET formation and inflammation in severe sepsis. Nat. Cardiovasc. Res..

[28] Larkin C.M., Santos-Martinez M.J., Ryan T., Radomski M.W. (2016). Sepsis-associated thrombocytopenia. Thromb. Res..

[29] Zhang Y., Peng W., Zheng X. (2024). The prognostic value of the combined neutrophil-to-lymphocyte ratio (NLR) and neutrophil-to-platelet ratio (NPR) in sepsis. Sci. Rep..

[30] Gonzalez D.A., Kumar R., Asif S., Bali A., Dang A.K. (2022). Sepsis and Thrombocytopenia: A Nowadays Problem. Cureus.

[31] Valet C., Magnen M., Qiu L., Cleary S.J., Wang K.M., Ranucci S., Grockowiak E., Boudra R., Conrad C., Seo Y. (2022). Sepsis promotes splenic production of a protective platelet pool with high CD40 ligand expression. J. Clin. Investig..

[32] Setarehaseman A., Mohammadi A., Maitta R.W. (2025). Thrombocytopenia in Sepsis. Life.

[33] Antcliffe D.B., Burrell A., Boyle A.J., Gordon A.C., McAuley D.F., Silversides J. (2025). Sepsis subphenotypes, theragnostics and personalized sepsis care. Intensive Care Med..

[34] Scherger S.J., Kalil A.C. (2024). Sepsis phenotypes, subphenotypes, and endotypes: Are they ready for bedside care?. Curr. Opin. Crit. Care.

[35] Qiu X., Li J., Bonenfant J., Jaroszewski L., Mittal A., Klein W., Godzik A., Nair M.G. (2021). Dynamic changes in human single-cell transcriptional signatures during fatal sepsis. J. Leukoc. Biol..

[36] Caraballo C., Jaimes F. (2019). Organ Dysfunction in Sepsis: An Ominous Trajectory From Infection To Death. Yale J. Biol. Med..

[37] Xu Z., Mao C., Su C., Zhang H., Siempos I., Torres L.K., Pan D., Luo Y., Schenck E.J., Wang F. (2022). Sepsis subphenotyping based on organ dysfunction trajectory. Crit. Care.

[38] Wang J., Blake L.M., Orozco N., Fiorini K., McChesney C., Slessarev M., Prager R., Leligdowicz A., Sharif S., Lewis K. (2025). Dynamic Measures of Fluid Responsiveness to Guide Resuscitation in Patients With Sepsis and Septic Shock: A Systematic Review and Meta-Analysis. Crit. Care Explor..

[39] Poston J.T., Koyner J.L. (2019). Sepsis associated acute kidney injury. BMJ.

[40] Pais T., Jorge S., Lopes J.A. (2024). Acute Kidney Injury in Sepsis. Int. J. Mol. Sci..

[41] Peerapornratana S., Manrique-Caballero C.L., Gómez H., Kellum J.A. (2019). Acute kidney injury from sepsis: Current concepts, epidemiology, pathophysiology, prevention and treatment. Kidney Int..

[42] Li J., Jia Q., Yang L., Wu Y., Peng Y., Du L., Fang Z., Zhang X. (2025). Sepsis-associated encephalopathy: Mechanisms, Diagnosis, and Treatments update. Int. J. Biol. Sci..

[43] Gao Q., Hernandes M.S. (2021). Sepsis-Associated Encephalopathy and Blood-Brain Barrier Dysfunction. Inflammation.

[44] Woźnica E.A., Inglot M., Woźnica R.K., Łysenko L. (2018). Liver dysfunction in sepsis. Adv. Clin. Exp. Med..

[45] Zarbock A., Koyner J.L., Gomez H., Pickkers P., Forni L. (2023). Acute Disease Quality Initiative Group. Sepsis-associated acute kidney injury-treatment standard. Nephrol. Dial. Transplant..

[46] Sonneville R., Benghanem S., Jeantin L., de Montmollin E., Doman M., Gaudemer A., Thy M., Timsit J.-F. (2023). The spectrum of sepsis-associated encephalopathy: A clinical perspective. Crit. Care.

[47] Johnson A.E.W., Bulgarelli L., Shen L., Gayles A., Shammout A., Horng S., Pollard T.J., Hao S., Moody B., Gow B. (2023). MIMIC-IV, a freely accessible electronic health record dataset. Sci. Data.

[48] Pollard T.J., Johnson A.E.W., Raffa J.D., Celi L.A., Mark R.G., Badawi O. (2018). The eICU Collaborative Research Database, a freely available multi-center database for critical care research. Sci. Data..

[49] Collins G.S., Reitsma J.B., Altman D.G., Moons K.G. (2015). Transparent reporting of a multivariable prediction model for individual prognosis or diagnosis (TRIPOD): The TRIPOD statement. BMJ.

[50] von Elm E., Altman D.G., Egger M., Pocock S.J., Gøtzsche P.C., Vandenbroucke J.P., STROBE Initiative (2008). The Strengthening the Reporting of Observational Studies in Epidemiology (STROBE) statement: Guidelines for reporting observational studies. J. Clin. Epidemiol..

[51] White I.R., Royston P., Wood A.M. (2011). Multiple imputation using chained equations: Issues and guidance for practice. Stat. Med..

[52] Zou G. (2004). A modified poisson regression approach to prospective studies with binary data. Am. J. Epidemiol..

[53] Dhainaut J.F., Shorr A.F., Macias W.L., Kollef M.J., Levi M., Reinhart K., Nelson D.R. (2005). Dynamic evolution of coagulopathy in the first day of severe sepsis: Relationship with mortality and organ failure. Crit. Care Med..

[54] Li J., Liu H., Wang N., Wang F., Shang N., Guo S., Wang G. (2024). Persistent high sepsis-induced coagulopathy and sequential organ failure assessment scores can predict the 28-day mortality of patients with sepsis: A prospective study. BMC Infect. Dis..

[55] Wang K., Lu D., Wang F. (2024). Subphenotypes of platelet count trajectories in sepsis from multi-center ICU data. Sci. Rep..

[56] Zhou H., Li Z., Liang H., Yan Z. (2022). Thrombocytopenia and platelet count recovery in patients with sepsis-3: A retrospective observational study. Platelets.

