# Supplementary material for: Dynamic Coagulation Deterioration and Cumulative Burden Preceding Noncoagulation Organ Dysfunction in Sepsis: A Two-Database Cohort Study
Source: J Clin Med. 2026 Oct 26;15(21):8326. doi: 10.3390/jcm15218326 (PMC13763603; doi:10.3390/jcm15218326)
Supplement: Supplementary file 1 [file jcm-15-08326-s001.zip › DCIR_SAMI_supplementary_figures_revised.pdf]

Figure S1. Dynamic coagulation index distributions

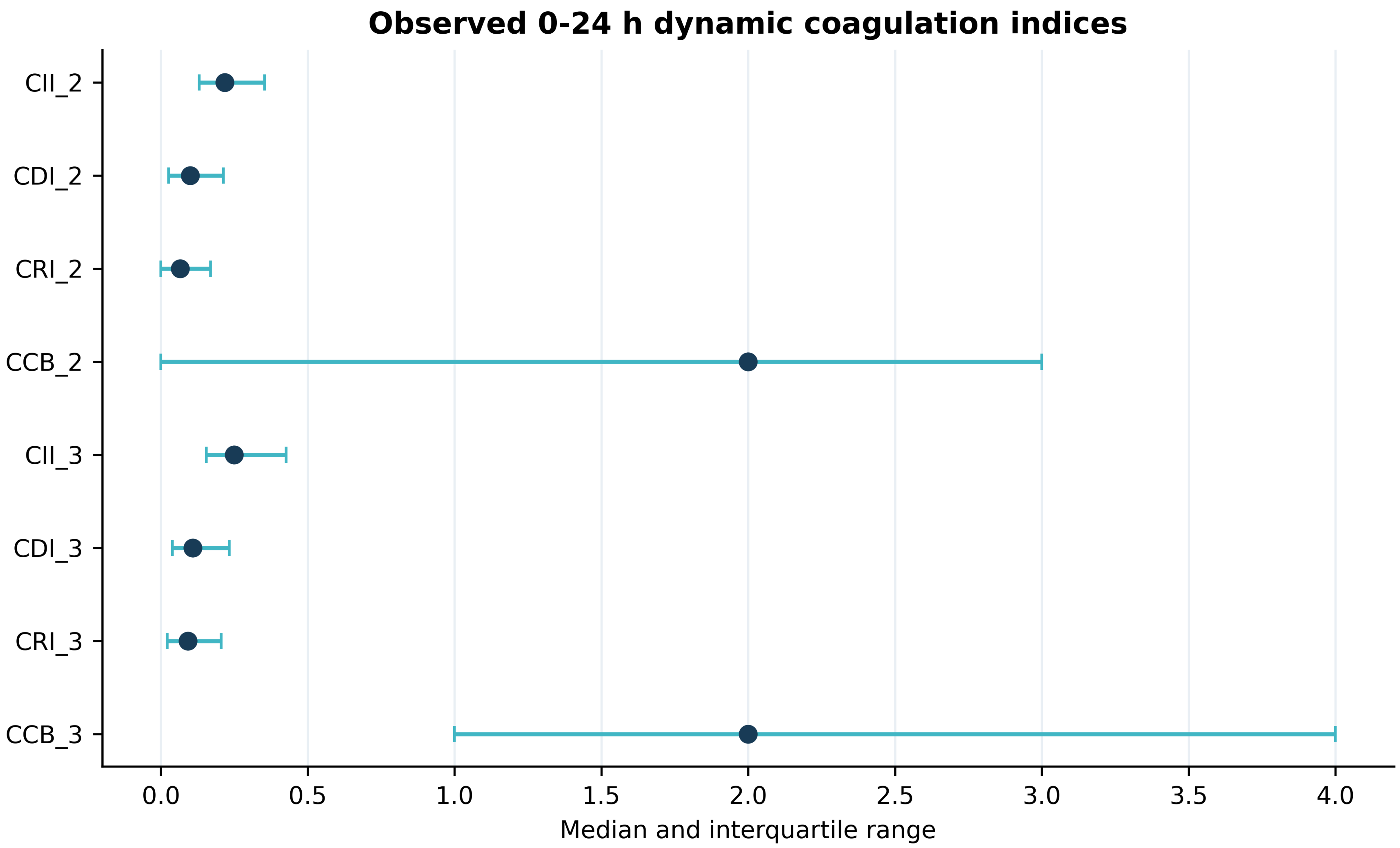

Observed 0–24-hour distributions of two-marker and three-marker dynamic coagulation indices. Points denote medians and bars denote interquartile ranges. Three-marker indices incorporate platelet count, INR, and PTT.

Figure S2. Temporal validation receiver operating curves

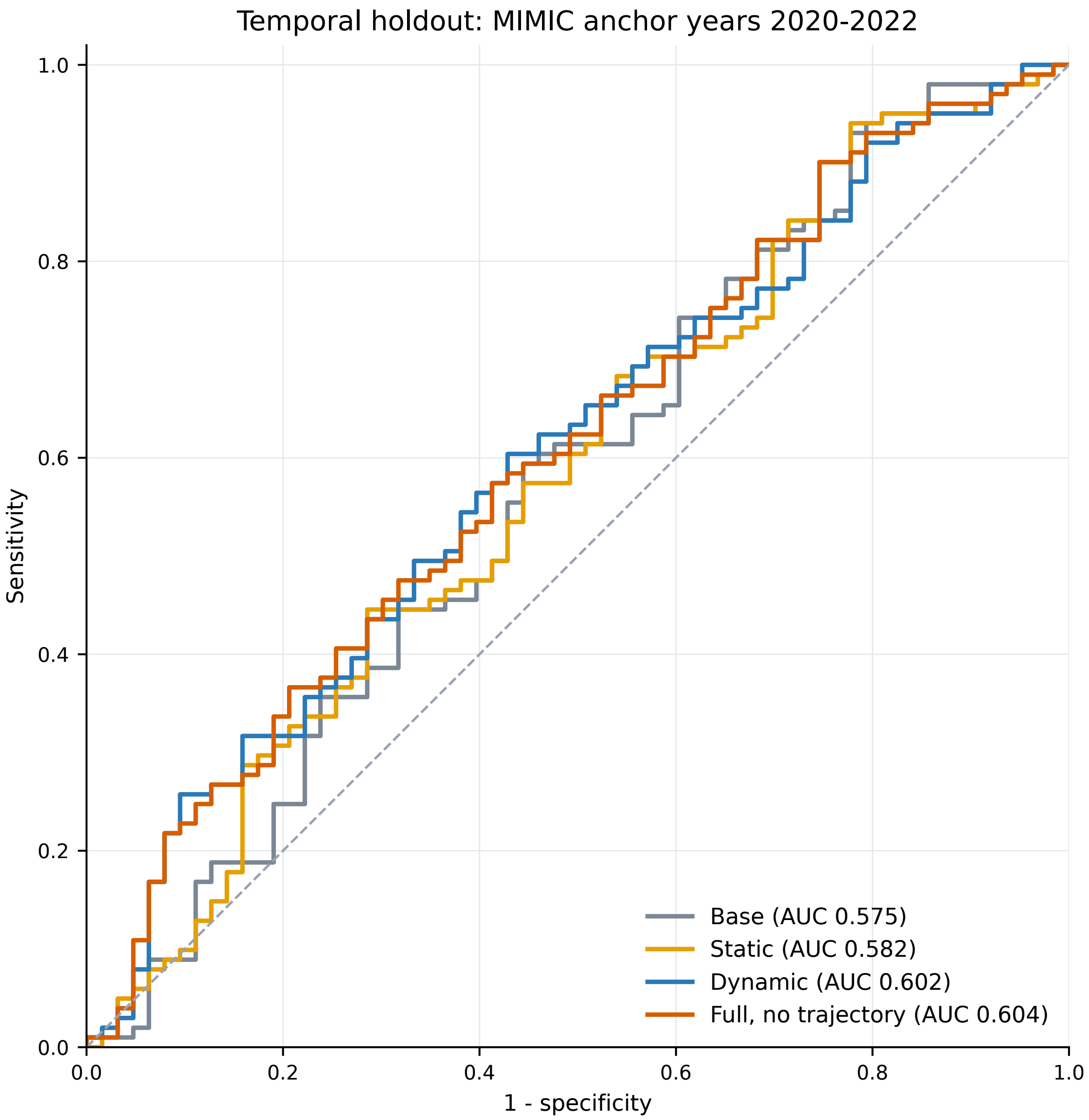

The holdout contained 164 stays and 101 events. Trajectory variables were excluded because their 24-72 h measurements overlapped the outcome window. Prediction analyses are supporting evidence; discrimination was modest.

Figure S3. Internal temporal validation and decision curves

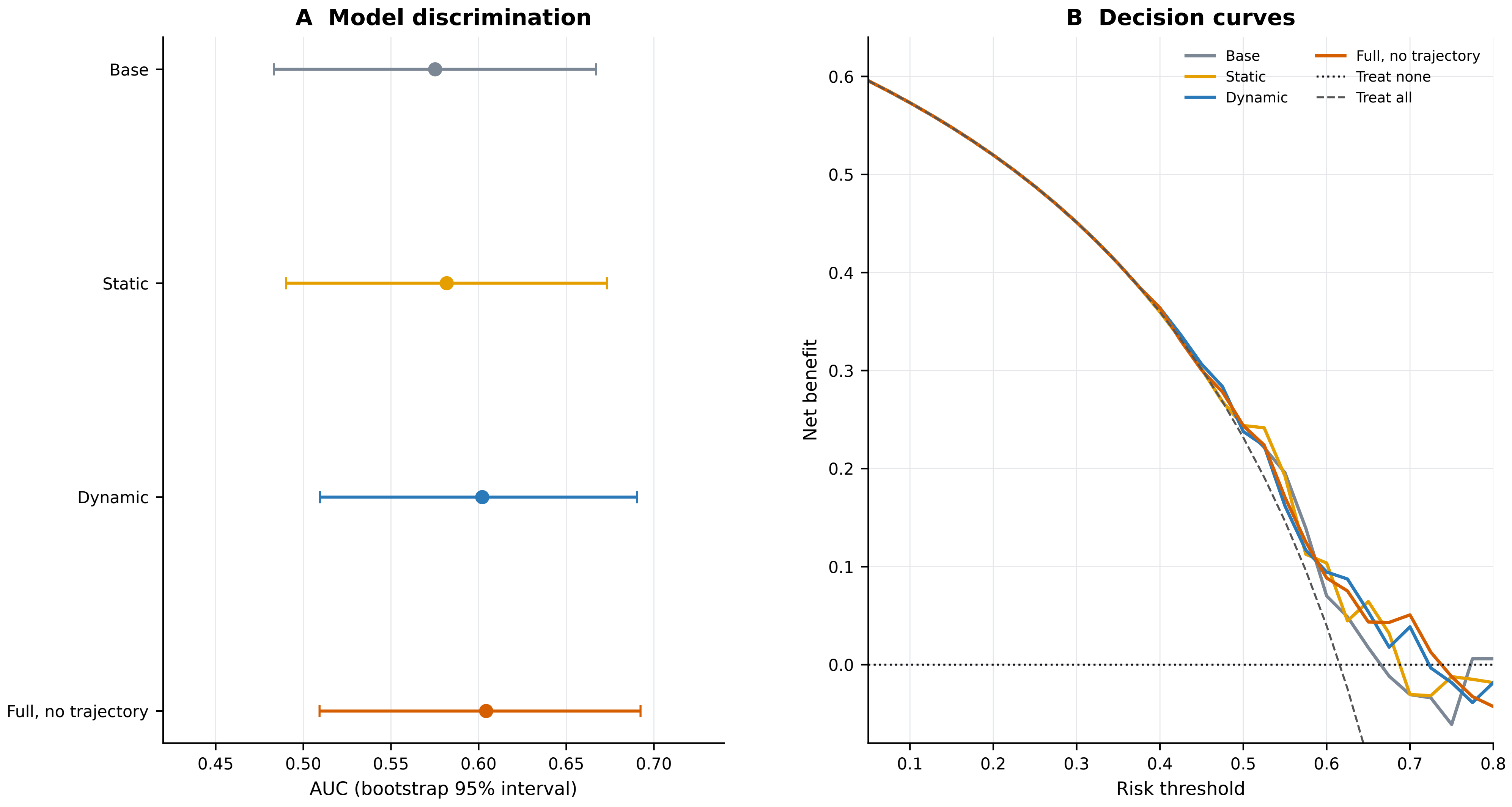

Models were fitted in MIMIC anchor years 2008-2019 and evaluated in 2020-2022. The full model excludes the overlapping 0-72 h trajectory variable. Broad bootstrap intervals and modest discrimination support use as an association/phenotyping analysis rather than a standalone prediction instrument.

Figure S4. Exploratory clinical subgroup associations

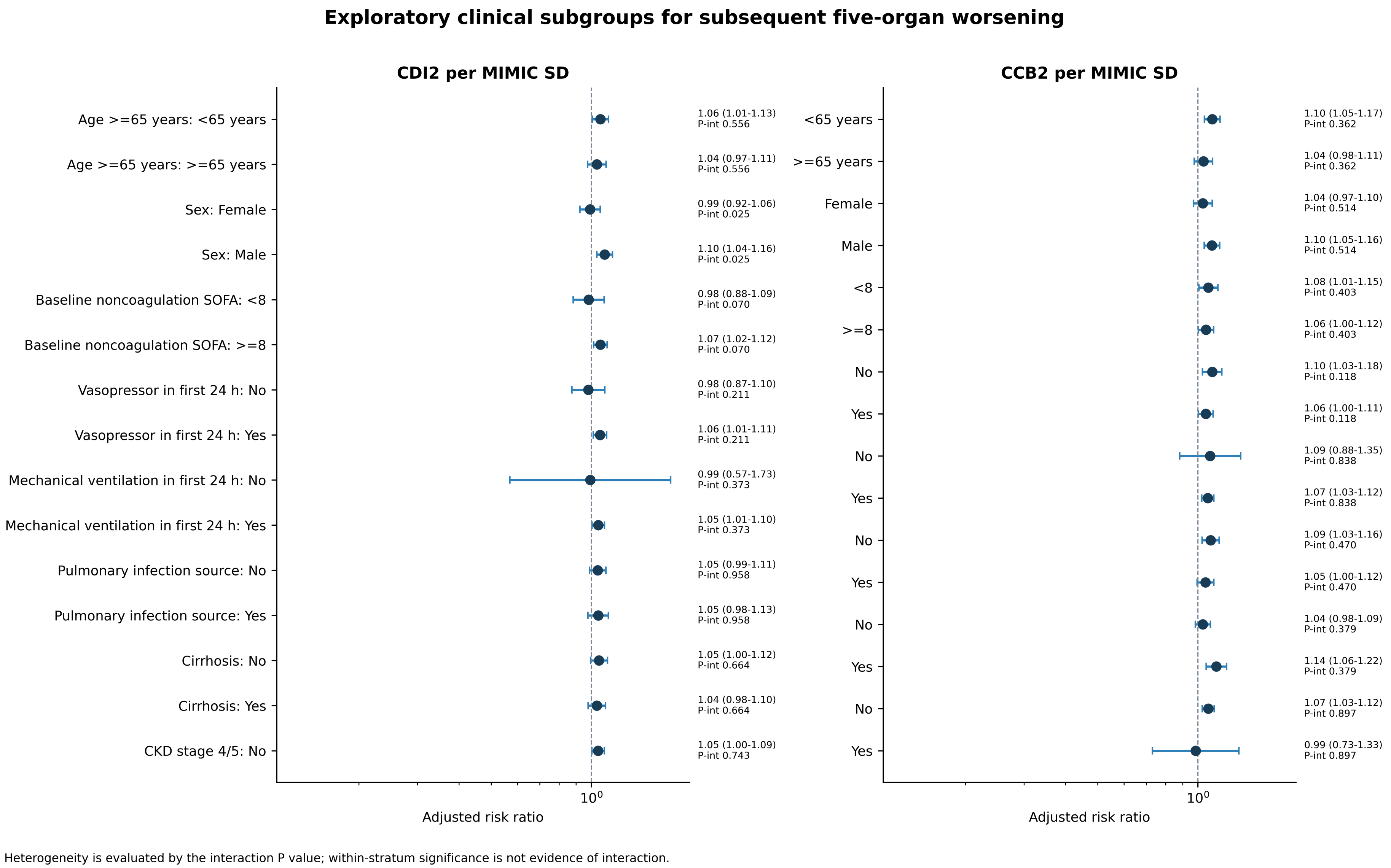

Adjusted risk ratios for the primary five-organ worsening composite across prespecified clinical subgroups. Heterogeneity is evaluated using the interaction P value; significance within a stratum does not establish interaction.

Figure S5. Adjusted high-burden organ associations

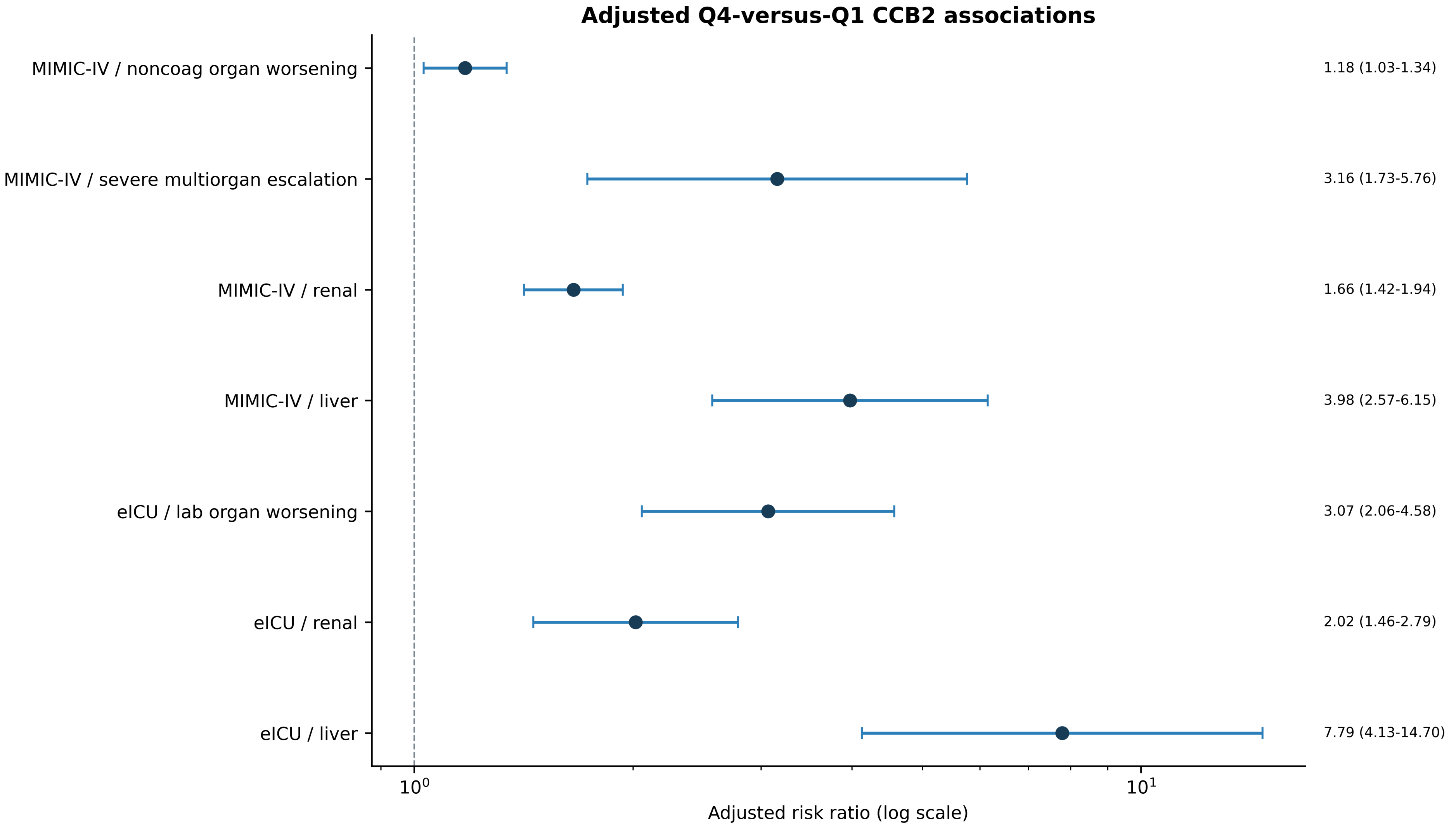

MIMIC-derived category boundaries were frozen for eICU. Estimates are associative and not causal.

Adjusted risk ratios comparing the highest versus lowest CCB2 category. MIMIC-IV category boundaries were frozen before application to eICU. Severe multiorgan escalation was an exploratory sensitivity outcome; all estimates are associative and noncausal.

Figure S6. Organ-specific CDI2 restricted cubic splines

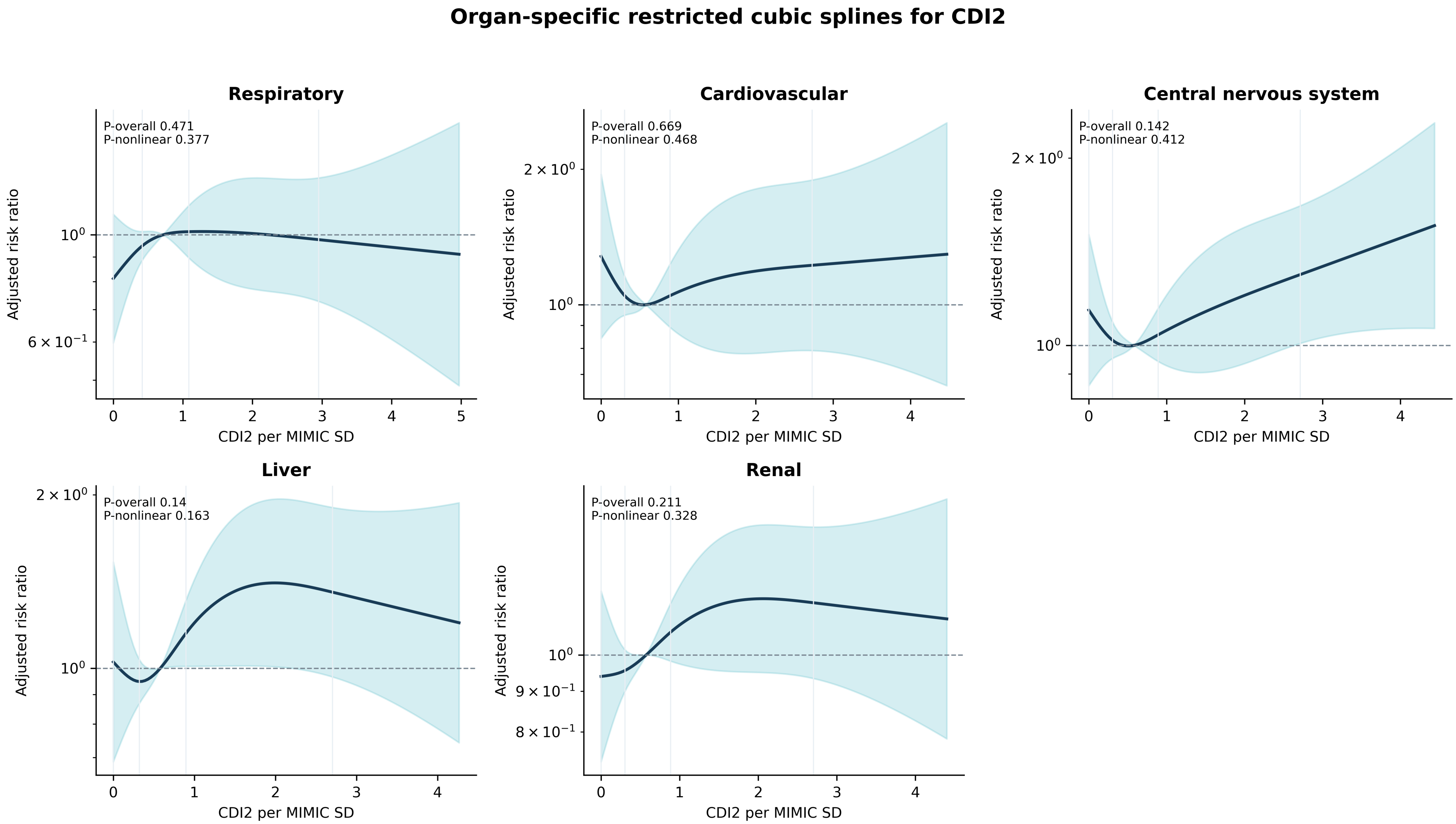

Curves use fixed Harrell knots and show adjusted risk ratios relative to the organ-specific median. Shading denotes 95% confidence intervals.

Adjusted restricted cubic spline curves for observed-care CDI2 and each organ-specific outcome. Four fixed Harrell knots were used. Curves are shown relative to the organ-specific median; every organ is retained regardless of statistical significance.

Figure S7. Organ worsening across CDI2 categories

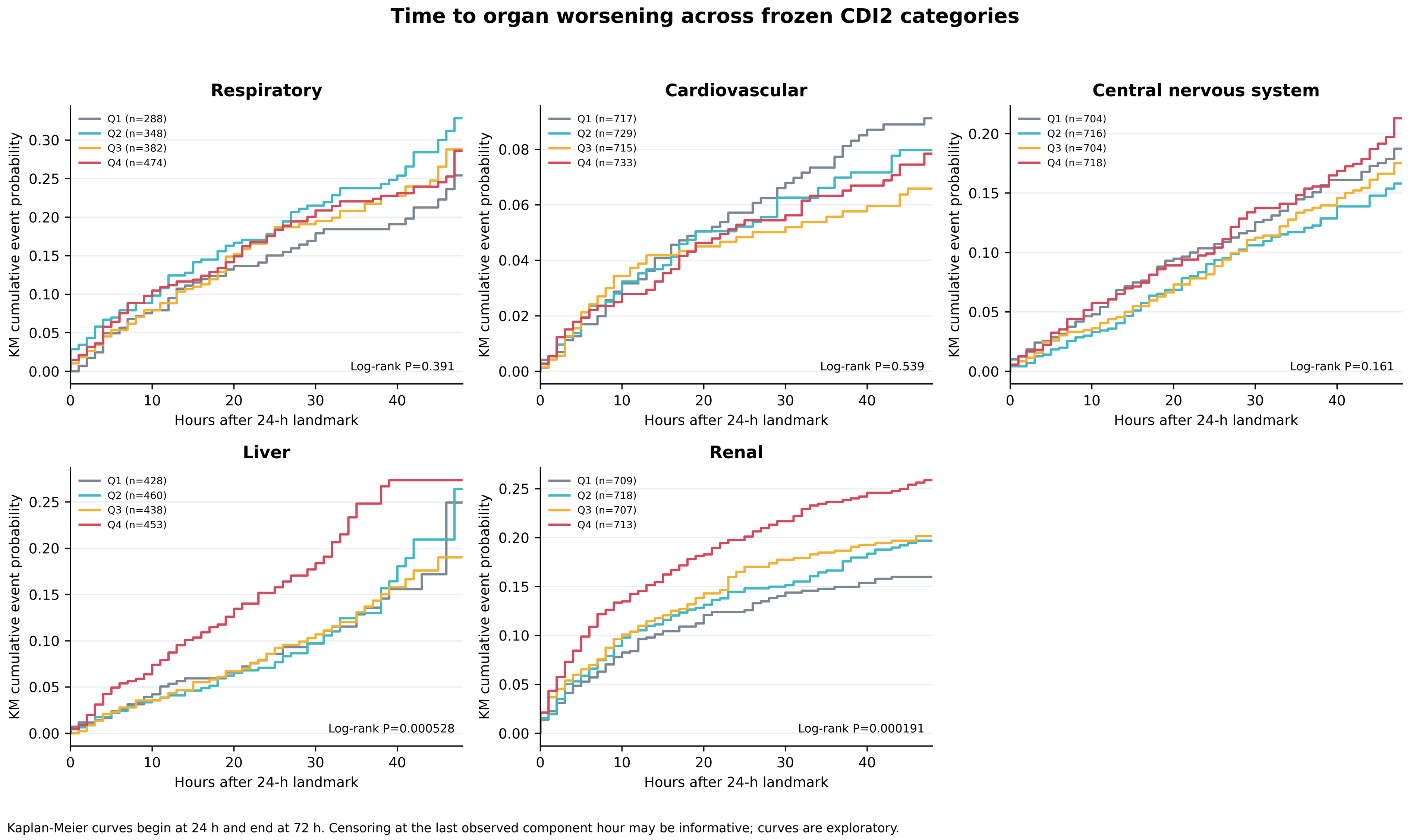

Kaplan-Meier-derived cumulative event curves from the 24-hour landmark through 72 hours across frozen CDI2 categories. Multigroup log-rank P values compare all four categories. Censoring at the final observed component hour may be informative.

Figure S8. Adjusted Cox models for first organ worsening

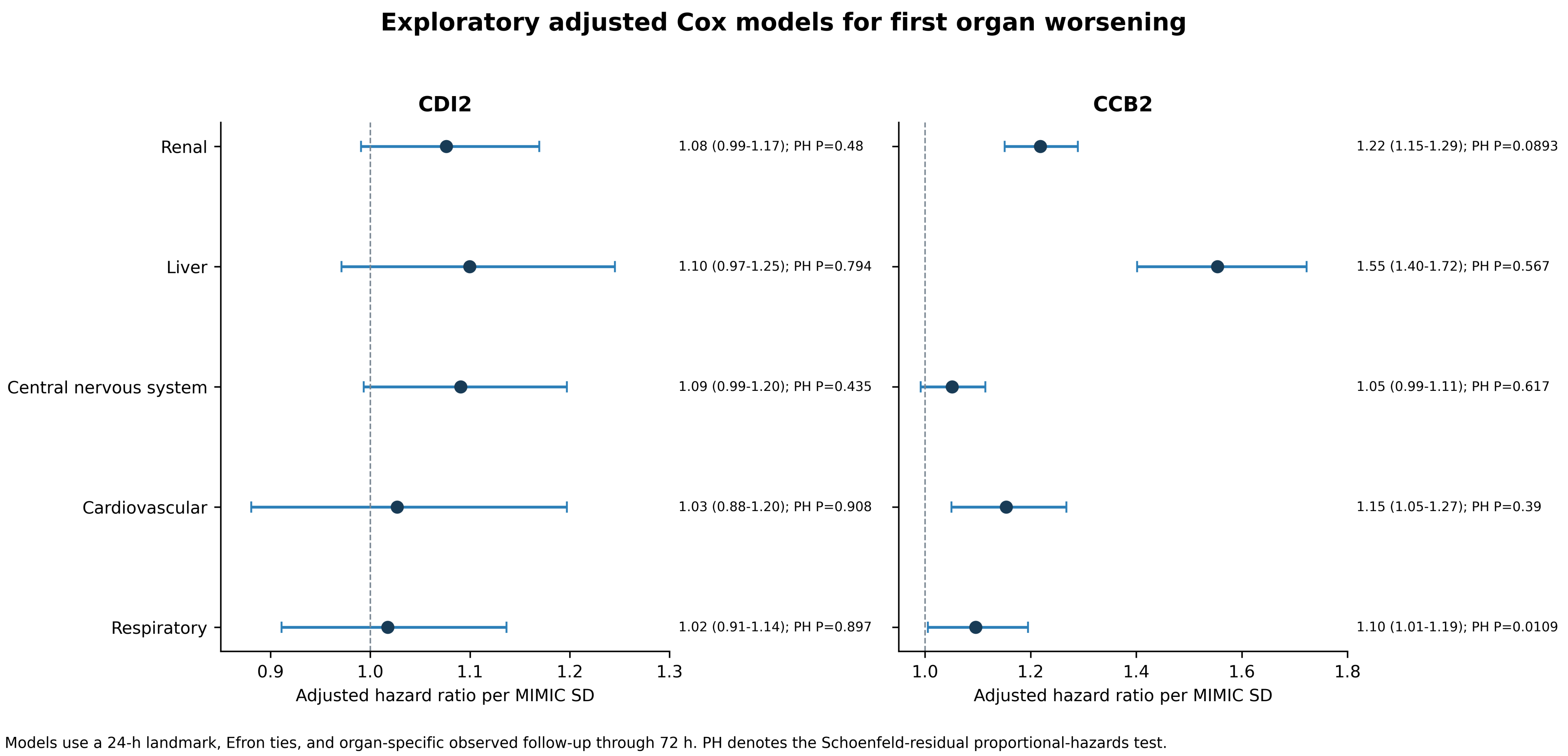

Exploratory adjusted hazard ratios per MIMIC standard deviation from the 24-hour landmark through 72 hours. Efron's method handled tied event times and covariates were multiply imputed. PH P values are Schoenfeld-residual time-trend tests; the respiratory CCB2 model did not satisfy the proportional-hazards assumption and its constant hazard ratio should be interpreted cautiously. Censoring at the final observed component hour may be informative.

Figure S9. Concurrent 0-72 h platelet-INR trajectory profiles

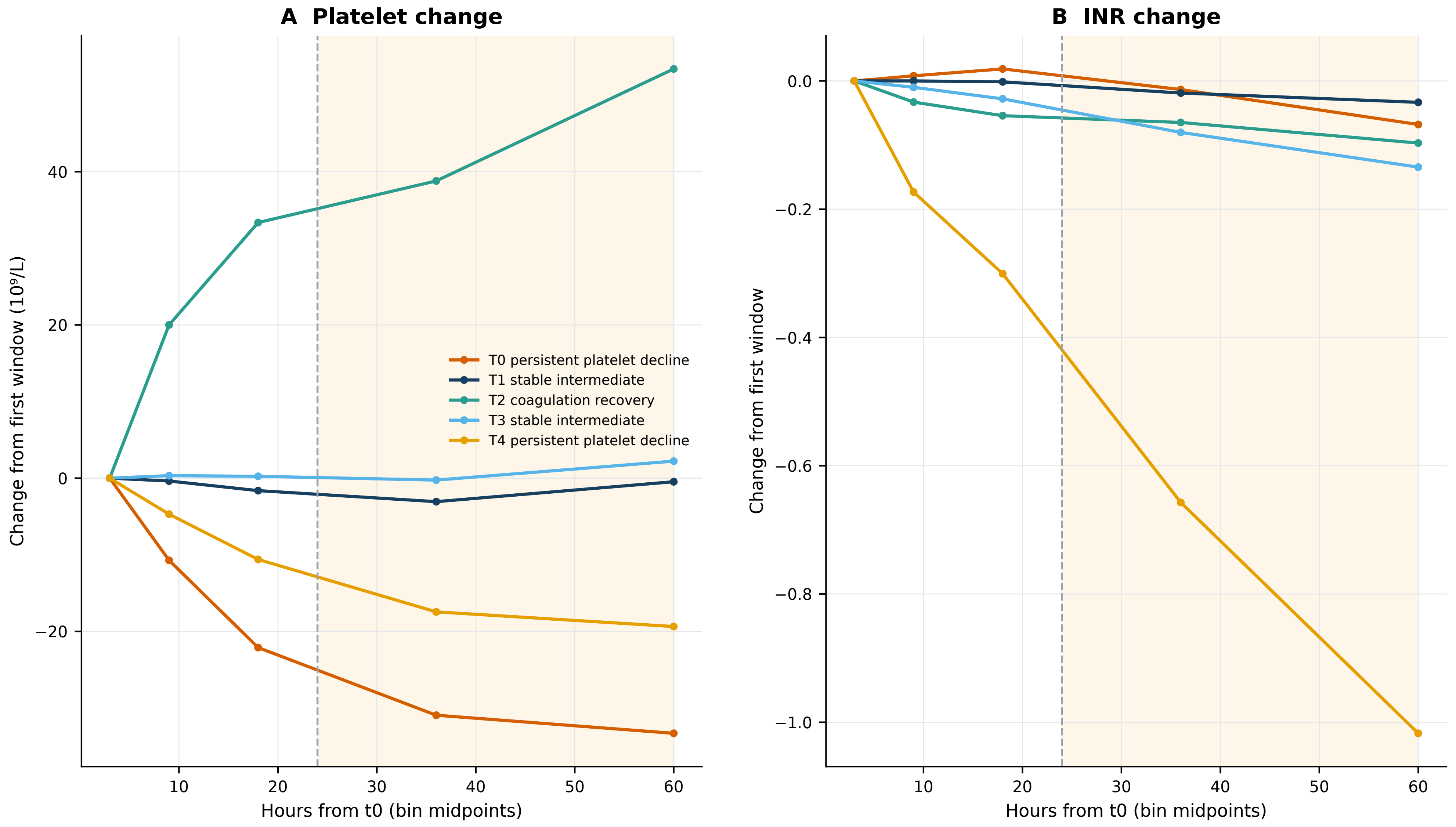

Profiles use five windows (0-6, 6-12, 12-24, 24-48 and 48-72 h; plotted at 3, 9, 18, 36 and 60 h). Shading marks overlap with the 24-72 h organ-outcome window. These classes are retained only as concurrent descriptive phenotypes and were excluded from temporally ordered association and prediction models.
