# Supplementary material for: Dynamic Coagulation Deterioration and Cumulative Burden Preceding Noncoagulation Organ Dysfunction in Sepsis: A Two-Database Cohort Study
Source: J Clin Med. 2026 Oct 26;15(21):8326. doi: 10.3390/jcm15218326 (PMC13763603; doi:10.3390/jcm15218326)
Supplement: Supplementary file 1 [file jcm-15-08326-s001.zip › supplementary_methods_revised.pdf]

Supplementary methods

Time ordering and analysis roles

Sepsis onset was time zero (t0) in MIMIC-IV. Dynamic coagulation exposures were calculated from measurements in 0-24 h, and organ outcomes were assessed from 24 h through 72 h; values at exactly 24 h belonged to the outcome window. The continuous two-marker coagulation deterioration index (CDI2) was the prespecified primary exposure. Coagulation instability (CII), recovery (CRI), cumulative coagulation burden (CCB), three-marker variants, organ-specific outcomes, severity gradients, dose-response analyses and event-time analyses were prespecified secondary or exploratory analyses. Prediction analyses were supporting analyses rather than the principal study aim.

Database-specific time zero

MIMIC-IV used reconstructed Sepsis-3 onset, whereas eICU-CRD used ICU admission because equivalent onset reconstruction was unavailable. In MIMIC-IV, the median interval from ICU admission to reconstructed t0 was 1.52 h. A sensitivity analysis recalculated CCB2 and the organ-outcome windows from ICU admission to emulate the eICU definition.

Fixed six-hour grid

Platelet count, international normalized ratio (INR) and partial thromboplastin time (PTT) measurements were assigned to fixed 6-h bins and summarized by the within-bin median. Duplicate specimen records were removed. No last observation carried forward was used. Change-based indices used only pairs of consecutive observed bins separated by exactly 6 h. CCB used only bins in which all markers required for that version were jointly observed.

Adverse orientation and robust standardization

For platelet count, lower values were adverse; for INR and PTT, higher values were adverse. Platelet values were used on the identity scale, whereas INR and PTT were log-transformed. Development-set medians and 1.4826 times the median absolute deviation (MAD) defined robust z scores, which were clipped to ±5 robust standard deviations. Multiplication by the prespecified adverse-direction sign produced z(m,b), the adverse-oriented score for marker m in bin b. These MIMIC development parameters were frozen and applied unchanged in eICU.

Change-index equations

For marker m, let A(m) denote its observed adjacent 6-h bin pairs and let $\Delta z(m,b)=z(m,b)-z(m,b-1)$. Marker-specific instability, deterioration and recovery were $I(m)=|A(m)|^{-1}\Sigma|\Delta z(m,b)|$, $D(m)=|A(m)|^{-1}\Sigma\max[\Delta z(m,b),0]$ and $R(m)=|A(m)|^{-1}\Sigma\max[-\Delta z(m,b),0]$, respectively. CII, CDI and CRI were the arithmetic means of I(m), D(m) and R(m) across the required markers. Consequently, CII=CDI+CRI. Each included marker required at least two valid bins and at least one adjacent pair. Two-marker versions used platelet count and INR; three-marker versions additionally used PTT.

Cumulative coagulation burden equation

CCB was an observed-bin mean, not trapezoidal area-under-the-curve integration. Platelet burden scores were 0 for ≥150, 1 for 100-149, 2 for 50-99 and 3 for <50 $\times 10^9$/L. INR burden scores were 0 for ≤1.2, 1 for 1.21-1.4, 2 for 1.41-1.8 and 3 for >1.8. PTT burden scores were 0 for ≤1.0, 1 for >1.0-1.5, 2 for >1.5-2.0 and 3 for >2.0 times the upper limit of normal. For patient i and eligible bin b, B2(i,b)=scorePLT(i,b)+scoreINR(i,b), and $CCB2(i)=|O2(i)|^{-1}\Sigma_{b\in O2(i)}B2(i,b)$, where O2(i) is the set of bins with both platelet and INR observed. CCB3 analogously added the PTT score and required all three markers in the bin.

Unobserved bins were excluded rather than assigned zero or interpolated. At least one joint bin was required; a sensitivity analysis required at least two joint platelet-INR bins.

Treatment-aware versions

The observed-care version used every eligible measurement in 0-24 h. The pre-direct-treatment version truncated the exposure window at the first platelet, plasma, cryoprecipitate, prothrombin complex concentrate, vitamin K, protamine or therapeutic anticoagulant event. The strict version additionally truncated at continuous renal replacement therapy (CRRT), extracorporeal membrane oxygenation (ECMO) or another major extracorporeal circuit. Prophylactic, therapeutic and circuit heparin remained separate. To distinguish truncation from changing eligibility, same-population analyses restricted observed-care estimates to patients also eligible for the corresponding strict index.

Concurrent trajectory description

The five-class platelet-INR trajectory model used the 0-6, 6-12, 12-24, 24-48 and 48-72 h windows, plotted at midpoints 3, 9, 18, 36 and 60 h. Because the last two windows overlapped the 24-72 h organ-outcome period, trajectory classes were retained only as concurrent descriptive phenotypes. They were excluded from all temporally ordered organ-association and prediction models. No claim of prospective prediction is based on these trajectories.

Organ outcomes and competing observation states

For each MIMIC Sequential Organ Failure Assessment (SOFA) respiratory, cardiovascular, central nervous system, liver and renal component, baseline was the maximum score in 0-24 h and outcome burden was the maximum in 24-72 h. An event required an outcome score ≥2 and an increase ≥1 point. The primary composite required all five components to be evaluable. Fixed-window observation was summarized separately for death during 24-72 h, ICU discharge

alive before 72 h and continued ICU stay at 72 h. Sensitivity analyses used (i) organ deterioration or death and (ii) any observed worsening when at least three components were evaluable, with adjustment for the number of evaluable components.

eICU component-level replication

Kidney and liver laboratory thresholds and documentation-based new vasoactive support were reproducible in eICU-CRD. Timed respiratory and central nervous system SOFA components were not sufficiently complete for the full five-organ composite. Therefore, eICU analyses constitute component-level replication using frozen MIMIC scales and category boundaries, not validation of the overall five-organ construct. The numbers of contributing hospitals were reported for every exposure-outcome analysis.

Covariates and multiple imputation

Exposures and outcomes were never imputed. Missing prespecified covariates were imputed in 20 chained-equation datasets with five iterative cycles and stochastic conditional-model draws; binary covariates were returned to 0/1 values. Estimates were combined with Rubin's rules. Troponin (58.5% missing) and albumin (64.6% missing) were displayed descriptively only and were excluded from the prespecified covariate tiers and from the imputation equations. Event-count information gates selected full, reduced or minimal covariate tiers.

Selection and measurement-process sensitivity analyses

Baseline characteristics, mortality and organ-outcome evaluability were compared among all 13,669 eligible stays, the 2,208 complete five-organ outcome cohort and the 1,002-patient primary CDI2 cohort using standardized mean differences. Stabilized inverse-probability-of-selection weights were estimated among patients alive at 24 h from age, sex, race, comorbidity, severity, infection source, organ support, anchor-year group, platelet/INR counts and overall

charting intensity, and were truncated at the 1st and 99th percentiles. Additional models adjusted for the raw numbers of platelet and INR measurements or for the numbers of valid 6-h bins. CCB2 was also stratified by one versus at least two joint platelet-INR bins.

Liver and time-zero sensitivity analyses

The liver association was re-estimated after excluding cirrhosis, after restricting baseline liver SOFA to <2, and after applying both restrictions. The ICU-admission-anchored MIMIC sensitivity used identical 0-24 h CCB thresholds and 24-72 h outcome rules relative to ICU admission, thereby matching the eICU time origin as closely as the available variables permitted.

Statistical interpretation and multiplicity

Modified Poisson models with robust standard errors estimated adjusted risk ratios. The continuous CDI2-primary-composite model was the prespecified primary association. No multiplicity adjustment was applied to secondary or exploratory analyses; organ-specific, categorical, spline, Kaplan-Meier, Cox, trajectory and subgroup P values are therefore descriptive. Interpretation emphasized effect sizes, 95% confidence intervals, coherence across sensitivity analyses and cross-database directional consistency.

Internal temporal validation

Models were developed in MIMIC anchor years 2008-2019 and evaluated in the 2020-2022 holdout. Base, static, dynamic and full-without-trajectory models were compared using the area under the receiver operating characteristic curve, precision-recall area, Brier score, calibration and decision curves. Trajectory predictors were excluded because they used measurements after 24 h. These analyses support, but do not define, the association study.

Restricted cubic splines and landmark event-time analyses

Restricted cubic splines, Kaplan-Meier curves and Cox models were exploratory. Four-knot spline models covered the 1st-99th percentiles and used the organ-specific median as reference. Landmark event-time analyses began at 24 h and censored event-free observations at the final observed component hour or 72 h. Because censoring may be informative, curves describe recorded event timing rather than biological latency or treatment benefit.

Respiratory proportional-hazards sensitivity

The continuous respiratory CCB2 Cox model violated the proportional-hazards assumption. A prespecified piecewise time-varying sensitivity estimated separate CCB2 hazard ratios for 24-48 h and 48-72 h and tested their interaction. This model is interpreted as evidence that the respiratory association may be stronger earlier in follow-up rather than as a single constant 24-72 h effect.

Quality control and reproducibility

Checks covered unique first ICU stays, exposure-outcome ordering, intervention truncation, post-unfractionated-heparin PTT, distinct blood-product categories, the CII identity, frozen eICU parameters, absence of exposure or outcome imputation, and exclusion of patient-level data from the public release. Supplementary Tables S24 and S25 report the added selection, observation-process, time-zero, hepatic and proportional-hazards sensitivity analyses.
